# Critical evaluation of key evidence on the human health hazards of exposure to bisphenol A

**DOI:** 10.3109/10408444.2011.558487

**Published:** 2011-03-25

**Authors:** JG Hengstler, H Foth, T Gebel, P-J Kramer, W Lilienblum, H Schweinfurth, W Völkel, K-M Wollin, U Gundert-Remy

**Affiliations:** 1 Leibniz Research Centre for Working Environment and Human Factors (IfADo), University of Dortmund, Dortmund, Germany; 2 Institute of Environmental Toxicology, University of Halle, Halle/Saale, Germany; 3 Federal Institute for Occupational Safety and Health, Dortmund, Germany; 4 Research and Development, Merck Serono, Darmstadt, Germany; 5 Dr. Lilienblum Consulting Toxicology LiCoTox, Hemmingen/Han, Germany; 6 Global Early Development, Bayer Schering Pharma AG, Berlin, Germany; 7 Bavarian Health and Food Safety Authority, Munich, Germany; 8 Lower Saxony Governmental Institute of Public Health, Hannover, Germany; 9 Federal Institute for Risk Assessment (BfR), Berlin, Germany

**Keywords:** Biomonitoring, endocrine disruption, estrogenic effects, intensive care units, interspecies extrapolation, low-dose effects, regulation, three- and two-generation studies, tissue deconjugation, tolerable daily intake, toxicokinetic modeling

## Abstract

Despite the fact that more than 5000 safety-related studies have been published on bisphenol A (BPA), there seems to be no resolution of the apparently deadlocked controversy as to whether exposure of the general population to BPA causes adverse effects due to its estrogenicity. Therefore, the Advisory Committee of the German Society of Toxicology reviewed the background and cutting-edge topics of this BPA controversy. The current tolerable daily intake value (TDI) of 0.05 mg/kg body weight [bw]/day, derived by the European Food Safety Authority (EFSA), is mainly based on body weight changes in two- and three-generation studies in mice and rats. Recently, these studies and the derivation of the TDI have been criticized. After having carefully considered all arguments, the Committee had to conclude that the criticism was scientifically not justified; moreover, recently published additional data further support the reliability of the two-and three-generation studies demonstrating a lack of estrogen-dependent effects at and below doses on which the current TDI is based. A frequently discussed topic is whether doses below 5 mg/ kg bw/day may cause adverse health effects in laboratory animals. Meanwhile, it has become clear that positive results from some explorative studies have not been confirmed in subsequent studies with higher numbers of animals or a priori defined hypotheses. Particularly relevant are some recent studies with negative outcomes that addressed effects of BPA on the brain, behavior, and the prostate in rodents for extrapolation to the human situation. The Committee came to the conclusion that rodent data can well be used as a basis for human risk evaluation. Currently published conjectures that rats are insensitive to estrogens compared to humans can be refuted. Data from toxicokinetics studies show that the half-life of BPA in adult human subjects is less than 2 hours and BPA is completely recovered in urine as BPA-conjugates. Tissue deconjugation of BPA-glucuronide and -sulfate may occur. Because of the extremely low quantities, it is only of minor relevance for BPA toxicity. Biomonitoring studies have been used to estimate human BPA exposure and show that the daily intake of BPA is far below the TDI for the general population. Further topics addressed in this article include reasons why some studies on BPA are not reproducible; the relevance of oral versus non-oral exposure routes; the degree to which newborns are at higher systemic BPA exposure; increased BPA exposure by infusions in intensive care units; mechanisms of action other than estrogen receptor activation; and the current regulatory status in Europe, as well as in the USA, Canada, Japan, New Zealand, and Australia. Overall, the Committee concluded that the current TDI for BPA is adequately justified and that the available evidence indicates that BPA exposure represents no noteworthy risk to the health of the human population, including newborns and babies.

## Contents

Abstract………263
Introduction………264
Derivation of tolerable daily intake values………265
Are the three-and two-generation studies of Tyl et al. valid?………265
Do oral low BPA doses below 5 mg/kg bw/day cause adverse health effects in laboratory animals?………267
How can differences between industry sponsored and publicly sponsored studies be explained?………270
Toxicokinetics………271
Humans………271
Non-human primates………275
Rats………275
Mice………275
Importance of the exposure route………275
Can rodents be used to extrapolate to the human situation with respect to estrogenic activity?………276
Are there susceptible subpopulations?………277
Toxicokinetics in children………277
Toxicokinetics in other groups………278
Tissue deconjugation of BPA-glucuronide and BPA-sulfate………278
Toxicodynamics………279
Specific exposure conditions………279
Neonatal intensive care unit………279
Polycarbonate (PC) bottles………279
How can biomonitoring support risk evaluation?………280
Results and interpretation of biomonitoring studies with BPA………280
Biomonitoring and exposure assessment………281
What are the mechanisms of action of BPA? Does the multitude of mechanisms besides estrogen receptor activation
make the substance more hazardous?………282
Epidemiological studies in the general population………283
Recent governmental responses………283
European Union………283
Denmark………284
France………284
Switzerland………284
Australia and New Zealand………284
Canada………285
USA………285
Japan………285
Acknowledgments………285
Declaration of interest………286
References………286

## Introduction

For more than 10 years there has been a scientific and journalistic controversy whether bisphenol A (BPA) causes adverse effects in humans related to its estrogenic activity (Borrell, 2010; Lorentzen and Hattan, 2010; Aschberger et al., 2010; Taylor et al., 2010; Yang et al., 2009). BPA, a building block of polycarbonate plastic and epoxy resins, was first synthesized in 1891. However, commercial production did not begin before the early 1950s when the first epoxy resins were developed (Vogel, 2009). Epoxy resins are extensively used as protective coatings on metal equipment, food cans, piping, and dental sealants. In 1957, it was discovered that polymerization of BPA with phosgene leads to polycarbonate. There is an unusual wealth of safety-related studies carried out on BPA. These cover nearly every possible endpoint. Its estrogenic properties were described as early as 1936 (Dodds and Lawson, 1936). To date, more than 5000 studies on BPA have been published. It is obvious that this should be enough information to resolve the controversy, but nevertheless this has not yet been achieved and those not directly involved in BPA research are usually puzzled by the never-ending and sometimes emotional debate. In order to contribute to a balanced and well-founded resolution of the seemingly deadlocked situation, the Advisory Committee of the German Society of Toxicology^1^ reviewed the background and the cutting-edge questions of the BPA controversy (Table 1) and offers an independent judgement.

**Table 1:** Cutting edge topics of the current controversy on BPA

Are the studies used for regulatory purposes flawed?
Do oral low doses below 5 mg/kg bw/day cause adverse health effects in laboratory animals?
How can differences between industry sponsored and publicly sponsored studies be explained?
Swallow or inject? What is the relevance of the oral route versus implantation of pumps or intravenous injection?
Can rodents be used to extrapolate to the human situation?
To which degree are embryos, babies, or children more susceptible?
How critical is exposure by intravenous infusion in intensive care units?
How critical is tissue deconjugation of BPA-glucuronide and BPA-sulfate?
How can biomonitoring support risk evaluation?
What are the mechanisms of action of BPA? Does the multitude of mechanisms other than estrogen receptor activation make the substance more dangerous?
Why are recent governmental responses inconsistent?

## Derivation of tolerable daily intake values

BPA has been tested in subchronic oral toxicity studies using rats, mice, and dogs (US EPA, 1984a, 1984b, 1984c; NTP, 1982). Rats and mice were administered BPA in the diet for 90 days (250–4000 ppm in rats; 5000–25,000 ppm in mice) (NTP, 1982). Doses higher than 1000 ppm (equivalent to approximately 67 mg/kg/day) lead to decreased body weight in both sexes of rats. In dogs (90 days; 1000–9000 ppm BPA in the diet), the only toxic effect observed was an increase in mean liver weight in the high-dose group (US EPA, 1984a). Bisphenol A was evaluated for developmental toxicity in CD rats (0, 160, 320, or 640 mg/kgbw/day) and CD-1 mice (0, 500, 750, 1000, or 1250 mg/kg bw/day) dosed daily by gastric intubation from gestational days 6 through 15. In Charles River rats, the only effect observed in two-generation studies (100–9000 ppm BPA in the diet) was decreased body weight in the F0 generation at 9000 ppm and in the F1 generation at doses equal or higher than 1000 ppm (US EPA, 1984b, 1984c). Also, male mice receiving doses higher than 15,000 ppm and the exposed females exhibited a decreased body weight gain compared to the controls (in mice 15,000 ppm is equivalent to approximately 1950 mg/kg/day based on a food factor of 0.13). In mice, doses of 1250 mg/kg/day led to maternal toxicity, including fetotoxic effects. However, no significant increase in the incidence of malformations was observed (NTP, 1985). In rats, doses equal to and higher than 1280 mg/kg/day were not toxic and did not cause malformations of the fetus (NTP, 1986).

Taken together, the toxic effects observed in laboratory animals after repeated BPA exposure occur at doses that are several magnitudes higher than the exposure of the general human population. Since it is not clear to which extent these rather early studies mentioned above accounted for internal quality assurance and were conducted according to accepted modern testing guidelines (or elements of them), their validity, reliability, and thus value for hazard and risk assessment are considered limited. Modern testing guidelines represent internationally agreed-upon guidance with the goal to provide a reproducible set of data that can optimally satisfy all internationally agreed safety assessment criteria used in the regulatory processes. Hence, the focus of this article is on findings of more recent studies conducted for regulatory purposes.

In a three-generation rat study (Tyl et al., 2002) and a two-generation study in mice (Tyl et al., 2008b, 2008c), BPA was found to decrease body weight, and the weights of the livers and kidneys. An overall no observed adverse effect level (NOAEL) of 5 mg BPA/kg bw/day was derived, based on liver weight decreases, the most sensitive end-point. At doses leading to liver weight changes, BPA did not cause any effects on hormone-sensitive endpoints, which are the focus of concern of the current debate on BPA toxicity. Using an uncertainty factor of 100 (10 for interspecies differences, 10 for interindividual differences), a tolerable daily intake (TDI) of 0.05 mg/kg bw/day was set by the European Food Safety Authority (EFSA, 2006) and confirmed in 2008 and 2010 (EFSA, 2008, 2010). This TDI has been accepted by most regulatory agencies worldwide. Similarly, the United States Environmental Protection Agency (US EPA) derived a reference dose of 0.05 mg/kg bw/day (reviewed by Willhite et al., 2008). Perhaps the most intensively currently discussed topic is whether the NOAELs used for deriving the TDI are scientifically valid and appropriate for risk assessment.

## Are the three- and two-generation studies of Tyl et al. valid?

The studies of Tyl et al. (2002, 2008b, 2008c) did not reveal any effects on fertility or development. Doses of 0.001, 0.02, 0.3, 5, 50, and 500 mg/kg bw/day of BPA were tested in CD Sprague-Dawley rats in a three-generation study (Tyl et al., 2002), and 0.003, 0.03, 0.3, 5, 50, as well as 600 mg BPA/kg bw/day in CD-1 mice in a two-generation study (Tyl et al., 2008b). The dose ranges in the latter studies also cover the “low-dose range.” Another two-generation reproductive toxicity study performed under good laboratory practice (GLP) did also not observe effects in the low-dose range (0.2–200 µg/kg bw/day) (Ema et al., 2001). However, criticism has been raised, e.g., by Myers et al. (2009), who described the studies of Tyl et al. (2002, 2008; Tyl 2009a, 2009b) as “so flawed as to be useless.” If these studies are considered invalid, this would have serious consequences indeed. Therefore, we collected the points raised by the critics and carefully considered their relevance. Considering the arguments summarized in Table 2, we came to the conclusion that all criticisms were already refuted convincingly by the author (R. Tyl) herself. In this context, it should also be considered that a further study published recently has confirmed a complete absence of effects over a wide range of BPA exposures in female rats exposed perinatally (Ryan et al., 2010a). In this study, BPA exposure 40-, 400-, or even 4000-fold higher than the maximum estimated exposure to humans in the general population caused no adverse effects (Ryan et al., 2010a; Sharpe, 2010). The endpoints in this study were adult sex hormone–dependent behavior and female reproductive development. The results are consistent with previously published studies that have shown an absence of reproductive effects in male rats and mice (for example: Tyl et al., 2002; Ema et al., 2001; Tinwell et al., 2002).

**Table 2:** Criticisms of the studies of Tyl et al. (2008a, 2008b, 2008c) and responses

Criticism	*Response*
1. Myers et al. questioned the use of CD-1 Swiss mice because of their “aberrant insensitivity” to estrogens.	Recently, Ryan et al. (2010a) used a wide range of doses from 0.05 to 50 µg/kg bw/day for the positive control estrogen, EE2. They observed a comparable sensitivity for CD-1 mice, Long-Evans, and Sprague-Dawley rats for several adverse effects. Therefore, the use of CD-1 mice by Tyl et al. (2008a, 2008b, 2008c) is justified. Tyl et al. purchased their CD-1 mice from Charles River to avoid supplier effects. It should be considered that also vom Saal and some of his colleagues used CD-1 mice and reported low dose effects (Timms et al., 2005). Low-dose effects of BPA were also reported in Long-Evans (Akingbemi et al., 2004) and in SD rats.
2. Myers et al. considered the prostate weights reported in the study of Tyl et al. (2008a, 2008b, 2008c) “abnormally high” and suggested “that the dissection procedures for the prostate in the Tyl laboratory included other nonprostatic tissues in the weight measurements, rendering them unusable.…” This criticism was also expressed by Gies et al. (2009).	Tyl (2009a, 2009b) reported that paraffin blocks and slides of the analyzed prostates are still available and indicate no evidence of extraneous tissue/fat or excessive inflammation (all recently audited by the US FDA). In a response letter (Tyl, 2009a, 2009b), the author documented that considering the age of the mice, prostate weights in their study were within the range of published data. Differences from vom Saal's study may be explained by his post wean caging regimen. Vom Saal's CF-1 mice were housed by sex. Pups were weaned at 23 days of age and male littermates were housed three per cage (Nagel et al., 1997). Prostate weight was determined when the mice were 6 months old. For this purpose, one male per litter was randomly selected and individually housed for 1 month before determination of prostate weights (Nagel et al., 1997). This procedure is problematic because the dominant cage male develops large androgen-dependent accessory organs, whereas subservient cage males have smaller prostates. It is questionable whether 1 month of single housing may compensate for months of group housing and its influence on sexual development of male mice. In the study of vom Saal (Nagel et al., 1997), no controls were presented with respect to the influence of group housing and compensation by single housing and the authors did not report whether they determined prostate weight only from the dominant males or if all animals were included. Since the authors wrote that “one male per litter was randomly selected…” (Nagel et al., 1997), they likely included both, dominant and subservient males. This may explain the high variability of the prostate weights in studies from vom Saal's lab compared to much lower variability in Tyl's studies (Tables 1 and 2 in Tyl, 2009a, 2009b). It should also be taken into account that the number of animals exposed to BPA in vom Saal's study (*n* = 7) is very low also, considering that an additional confounder (dominant versus subservient mice) may be present. Much higher numbers of animals we re used in Tyl's studies (28 per group). After careful evaluation of Tyl's studies, the criticism of Myers et al. (2009) and Gies et al. (2009) concerning prostate weights is unsubstantiated. However, several aspects may be critical in vom Saal's studies, such as the influence of dominant versus subservient males, no available histology, and use of a now obsolete in-house strain preventing other researches to reproduce the initial positive studies. Another drawback is the statistical analysis.Concerning the criticism that crude mistakes were made by Tyl's laboratory in determining prostate weights, it should also be considered that this laboratory has carried out a large number of reprotox studies (often sponored by the US Government) and has successfully joined interlaboratory validation studies, where the Tyl laboratory achieved some of the most precise prostate weight data and robust treatment-related effects (EPA review).
3. Myers et al. criticized the high incidence and severity of prostatitis in the animals from Tyl's study, which may compromise their results.	The paraffin slides of the prostates of the mice from Tyl's study are still available and have been reanalyzed by an independent pathologist. The results have been published (Tyl, 2009a, 2009b). The incidence of inflammation in CD-1 mice of Tyl's study matches the low incidences of prostatitis seen in many mouse strains without treatment. Therefore, it is extremely unlikely that the results of Tyl et al. (2008a, 2008b, 2008c) have been compromised by prostatitis. It should be kept in mind that vom Saal's laboratory did not include any histopathological examinations (Nagel et al., 1997). Therefore, a possible confounding influence of prostatitis could not be accounted for in their study.
4. Myers et al. criticized that the diet used in Tyl's study contained phytoestrogens, which they claim would interfere with BPA activity.	Recently, Tyl et al. have published the genistein, daidzein, and glycitein contents of the standard diet (Purina Certified Ground Rodent Chow No. 5002) used in their study. Vom Saal and colleagues have not reported the phytoestrogen content of their diet. The majority of “normal rodent diets have similar levels of phytoestrogens.” Although it is not possible to compare diets between Tyl's and vom Saal's laboratories, it is extremely unlikely that the diet in Tyl's study compromised an estrogenic response, because in studies with estradiol in which mice were fed the same standard diet as those in the BPA studies, estradiol (0.5 ppm) clearly accelerated acquisition of puberty (Tyl, 2008a).
5. Myers et al. criticized that “Tyl et al. (2008a) did not examine any neurobehavioral end points,” which Myers et al. interpreted as a “glaring omission of Tyl.”	The multigeneration study performed by Tyl et al. (2008a) represents a standard test performed according to a specific guideline that is not designed to investigate neurobehavioral endpoints. Therefore, there is certainly no “glaring omission of Tyl,” because behavior simply is not an endpoint in this study type. However, there are other studies that have focussed on neurobehavioral endpoints: Ema et al. (2001) observed no effects in their two-generation, low-dose (0.2–200 μg BPA/kg bw/day) study. Similarly, Ryan et al. (2010a) obtained negative results in a detailed study on reproductive development, function, and behavior in female rats exposed perinatally to a wide range of BPA doses (see also Sharpe, 2010). Stump et al. (2010) published the most recent study conducted according to GLP. The results show that BPA, at levels of exposure 4000-fold higher than the maximum human exposure in the general population, does not cause any discernible adverse effects in female rats. By contrast, EE2, used as a positive control in this study, caused major adverse health effects at doses in the range of those applied in early contraceptives (Ryan et al., 2010a; highlight report: Sharpe, 2010).
6. Myers et al. criticized Tyl et al. (2008a, 2008b, 2008c) for using too many animals: “…all of the studies by Tyl et al. were significantly overpowered and this is in direct violation of federal guidelines for conducting animal research, a fact about which U.S. FDA regulators seem unaware.”	The consideration of animal protection aspects should certainly be an important point in all animal testing. But even if we assume that more animals than required were used, according to statistical power analysis, this would not lead to different or incorrect conclusions from a study. However, the numbers of animals used in Tyl's studies are appropriate—28 mice/sex/group/generation. This is in line with the OECD and US EPA toxicity testing guidelines, which require at least 20 pregnant females per group.

The argument of Myers et al. (2009), claiming that estrogen-insensitive mouse and rat strains have been used by Tyl et al (2002, 2008b, 2008c), has to be judged as not valid. As pointed out by Ryan et al. (2010b), CD-1 mice respond to low doses of exogenously administered estrogens, as do the rat strains used by these authors (Ryan et al., 2010b). Nagel et al. (1997), in vom Saal's laboratory, reported enlarged prostates in F1 adult CF-1 mice from in utero exposure to 2 and 20 µg/kg bw/day oral BPA. These results could not be confirmed by other investigators who tried to reproduce the study with higher numbers of animals (Cagen et al., 1999; Ashby et al., 1999). Unfortunately, vom Saal's laboratory has meanwhile closed their colony of CF-1 mice, which precludes reproduction of the study. Therefore, the question remains open as to whether this strain was particularly sensitive to BPA or whether the positive result was an artefact.

We addressed the criticism of Gies et al. (2009) that “Also no reason was found why the GLP-studies (Tyl et al., 2002, 2008; Tyl, 2003) used for regulatory purposes (by European authorities) did not find BPA effects and whether the reported prostate weights are inconsistent with literature data.” The most obvious explanation for the lack of BPA effects is that the compound simply does not cause any adverse health effects in the low-dose range. Also, the prostate weights reported by Tyl et al. (2002, 2008) are certainly not inconsistent with literature data. Considering age and caging schedules, the prostate weights of interest are in agreement with published literature (Tables 1 and 2 in Tyl, 2009a, 2009b). Data have been obtained in a commercially available mouse strain and can therefore easily be reproduced.

In conclusion, the criticism of Myers et al. (2009) and Gies et al. (2009; see also comments to this paper at: http://www.umweltbundesamt.de/gesundheit-e/veran-staltungen/bisphenol-a/index.htm) regarding the value of the three- and two-generation studies of Tyl et al. are unsubstantiated.

## Do oral low BPA doses below 5 mg/kg bw/day cause adverse health effects in laboratory animals?

Several guideline-compliant toxicity studies have been performed that resulted in a systemic NOAEL of 5 mg BPA/kg bw/day and a reproductive/developmental NOAEL of 50 mg BPA/kg bw/day in rats and mice (Table 3). Consistently, no adverse health effects were observed at doses ≤5 mg BPA/kg/day.

**Table 3:** Examples of guideline-compliant BPA studies

Study	Design	Result	Reference
Three-generation reproductive toxicity study in rats	• Six BPA dose groups (0.001–500 mg/kg/day; administration in diet);	• No effects in the low- dose range (0.001–5 mg/kg/day)	Tyl et al., 2002
	• OPPTS guideline 870.03800	• Reduced body and organ weights at ^3^50 mg/kg/day	
		• Effects on renal and hepatic histopathology: 500 mg/kg/day	
		• Reproductive and developmental toxicity: 500 mg/kg/day	
		• At doses <500 mg/kg/day: no effects on prostate weights, acquisition of puberty	
		• Systemic NOAEL: 5 mg/kg/day	
		• Reproductive NOAEL: 50 mg/kg/day	
Two-generation rat study	• Daily gavage doses of 0.0.2–200 µg/kg/day	• *No* effect at any dose (consider that this is a low-dose study with 0.2 mg/kg/day as the highest dose)	Ema et al., 2001
	• US EPA GLPs and OPPTS TG with added endocrinesensitive and neurobehavioral end points		
One- and two-generation reproductive toxicity studies in mice	• Two vehicle control groups, six BPA dose groups (0.003–600 mg/kg/day; administration in diet)	• No effects in the low-dose range (0.003–5 mg/kg/day)	Tyl et al., 2008a, 2008b, 2008c
	• Dietary estradiol as positive control (0.2 µg/kg/day to 8 mg/kg/day)	• Reduced body weights, increased renal and liver weights and further effects at 600 mg/kg/day. However, no adverse effects on adult reproductive structures or functions at 600 mg/kg/day.	
	• OECD GLPs		
		• Effects on liver histopathology at 50 mg/kg/day	
		• Systemic NOAEL: 5 mg/kg/day	
		• Reproductive NOAEL: 50 mg/kg/day	
Developmental neurotoxicity study in rats	• Dietary administration of 0, 0.01, 0.1, 5, 50, and 150 mg/kg/day (mean target doses) from gestation day 0 through lactation day 21. Evaluation of F1 offspring.	• No treatment-related neurobehavioral effects	Stump et al., 2010
	• OECD test guideline 426	• No evidence of neuropathology and no effects on brain morphometry	
		• NOAEL for systemic toxicity derived from maternal and offspring body weight reductions: 5.85 and 13.1 mg/kg/day (calculated) during gestation and lactation, respectively	

In addition to these guideline-compliant studies, a high number of exploratory research studies have been performed that usually study early, molecular, or other endpoints, of which the relevance for adverse health effects often has not yet been validated. If these studies are reproducible and their interpretation is clear, they should be used to support risk assessment. However, conclusions from explorative research studies on BPA were inconsistent (reviewed in Goodman et al., 2006; 2009; Gray et al., 2004; Willhite et al., 2008; Chapin et al., 2008; EFSA, 2006). For example, vom Saal's laboratory performed a study in F1 adult mouse offspring (CF-1 mice originally obtained from Charles River and later maintained as an outbred colony in the authors' facility), which were orally dosed with BPA at 2 and 20 μg/kg bw/day on gestational days 11–17 (Nagel et al., 1997). The authors reported similar increases in prostate weights in both exposure groups (2 and 20 µg/kg bw/day) of 30–35% compared to controls. However, this result could not be reproduced in later studies with higher statistical power, for example by Cagen et al. (1999) and Ashby et al. (1999). The negative outcomes in the studies of Cagen et al. (1999) and Ashby et al. (1999) were later criticized by vom Saal and colleagues (Myers et al., 2009), because they were not considered useful for risk assessment by the NTP-CERHR (National Toxicology Program Center for the Evaluation of Risks to Human Reproduction). However, the studies of Cagen et al. (1999) and Ashby et al. (1999) had been designed to repeat and verify vom Saal's study with larger numbers of animals. They were not intended for regulatory purposes and, therefore, did not follow the protocol of a toxicity testing guideline. Possible reasons for the non-reproducibility of vom Saal's study are the small numbers of only seven mice per dose groups and the lack of control of the confounding effect of dominant versus subservient mice on prostate weights during group housing (Table 2, criticism 2).

In a situation with apparently contradictory data, it is prudent to have the complete set of data evaluated by a panel of experts (Chapin et al., 2008; EFSA, 2006, 2008, 2010a, 2010b; Goodman et al., 2006, 2009; US FDA Memorandum, 2009a, 2009b; Willhite et al., 2008). Therefore, the NTP-CERHR formed such an expert panel, including several internationally known scientists working on BPA, toxicologists, epidemiologists, and statisticians (NTP, 2008). This expert panel has evaluated more than 700 studies trying to extract possible evidence of adverse health effects. Many of the studies included in the assessment failed to meet minimal quality criteria for experimental design and statistical analysis.^2^ Among the most common deficiencies was a failure to control for “litter effects,” although litter-based statistics have been specified by the US EPA. Siblings from the same litter often have similar properties. If this is not considered in the study design, random variation between litters may be misinterpreted as a signal of a treatment-related effect. The expert panel also did not consider studies that did not include a concurrent control group of animals, injected BPA into the brain or spinal cord, or contained positive control groups that did not show adverse effects. Finally, the NTP expert panel applied the five possible levels of concern (negligible concern, minimal concern, some concern, concern, and serious concern) and concluded that there was “some concern” for BPA-associated effects on the brain, behavior, and prostate, whereas most other effects were rated as of “negligible” or “minimal” concern (NTP, 2008). Consequently, further studies were designed to clarify the situation where the NTP expert panel expressed “some concern.” One example is the study by Ryan et al. (2010a) in which pregnant Long-Evans rats were treated orally by gavage with 0, 2, 20, and 200 µg BPA/kg bw/day from day 7 of gestation to postnatal day 18. In the female offspring that were examined, BPA did not alter sexually dimorphic behavior, puberty, fertility, or anatomy (Ryan et al., 2010a). In the same study, ethinyl estradiol (EE2) at doses of 0.05–50 µg/kg bw/day increased anogenital distance, reduced pup body weight, accelerated age at vaginal opening, reduced F1 fertility and F2 litter sizes, and induced malformations of the external genitalia (Ryan et al., 2010a). In a second study, BPA (2, 20, or 200 µg/kg bw/day) was administered by oral gavage during pregnancy from gestational day 7 to postnatal day 18 and the male offspring were studied (Howdeshell et al., 2008). BPA did not affect androgen-dependent reproductive organ weights, including prostate weights or epididymal sperm abundance. By contrast, adult body weight was reduced by EE2 at 50 µg/kg bw/day and androgen-dependent tissue weights were reduced in a dose-dependent manner (Howdeshell et al., 2008). Therefore, these new studies have addressed some of the endpoints about which the NTP expert panel had expressed “some concern.” The negative outcome of these large and well-designed studies prompted the question as to whether it is time to end concerns over the estrogenic effects of BPA, particularly since it has repeatedly been impossible to reproduce the initial positive effects (Sharpe, 2010).

The difficulties surrounding the discussions on low-dose effects of BPA are illustrated by a study performed by Schönfelder et al. (2002a). This study is often cited to support the opinion that low-dose BPA exposure alters the development of estrogen-sensitive organs in rodents (for example: Somm et al., 2009; Kum et al., 2009; Newbold et al., 2009; Vandenberg et al., 2008; Vom Saal and Welshons, 2006). Interestingly, this study has been classified as inadequate for the evaluation process by an expert panel (Chapin et al., 2008). Therefore, we intensively revisited the controversially discussed study of Schönfelder et al. (2002a) in which Sprague-Dawley dams were administered 0.1 and 50 mg/kg bw/day BPA from gestation days 6 to 21 (Schönfelder et al., 2002a). The authors report that they observed “striking morphological changes” in the vagina of postpubertal offspring” and “that the full-length ERalpha is not expressed during estrus in the vagina of female offspring.…” The “thickness of the total epithelium was reduced…” following exposure to 0.1 mg/kg BPA (Figure 1B in Schönfelder et al., 2002a) when compared to the control group (Figure 1A in Schönfelder et al., 2002a). The 50 mg/kg dose caused a similar effect (as the 0.1 mg/kg dose), but this was less pronounced (Figure 1D in Schönfelder et al., 2002a). Taking these results very seriously, we, however, have to report that severe criticism has been expressed (Chapin et al., 2008) arguing that the study of Schönfelder et al. (2002a) does not give any information on the numbers of animals used and the number of offspring examined. Also, it lacks any statistical analysis of the results. Even the number of litters represented was not stated. It is important that the litter and not the individual rodent pup be used for statistical analysis, because there may be large differences between individual litters (Goodman et al., 2006; Willhite et al., 2008). It is a critical difference if the “six offspring in the 50 mg/kg per day BPA group” (Schönfelder et al., 2002a) are pups from six separate litters rather than from only one litter. This has not been specified by Schönfelder et al. (2002a). The study was criticized by the EFSA (2006) because the results (Schönfelder et al., 2002a) were taken from experiments where none of the control and BPA treatments were performed at the same time. Therefore, the EFSA expert panel came to the conclusion that this casts doubt on the robustness of the observations and makes this study unsuitable for risk assessment (EFSA, 2006). After our own thorough assessment of this study we came to the same conclusions as the expert panel mentioned above (Chapin et al., 2008). This means that according to the rigorous requirements in the regulatory process, we have to conclude that the data of this study do not meet the criteria necessary for a regulatory safety assessment.

Because of the high public interest into the question whether low-dose BPA may cause adverse effects and based on quality issues with some studies claiming important adverse effects, the National Institute of Environmental Health Sciences (NIEHS) has just announced an investment of approximately 30 million US dollars into BPA-related research (Spivey, 2009), including 10 two-year studies on the potential contribution of low-dose BPA to obesity, diabetes, reproductive disorders, asthma, sexually dimorphic behaviors, cardiovascular diseases, as well as prostate, breast, and uterine cancer. Although BPA is already one of the most intensively studied chemical compounds, the new wave of studies will offer the chance to achieve an even clearer picture and to provide the basis for a final decision on the existing controversy. As part of the “lessons learnt,” we come to the conclusion that in all future studies, as a minimum requirement, the following, principally self-evident, rules should be followed: (i) study design, endpoints, and statistical procedures must be defined in a study plan before the onset of the study; (ii) sufficient numbers of doses should be tested (studies with only one or two doses are not sufficient) and the number of animals per group, accounting also for litter effects, must be sufficient according to statistical power analyses; (iii) validated or clearly interpreted endpoints should be tested; (iv) the route of exposure should be known for the exposure routes in humans; (v) study plan and the obtained raw data should be transparent and made available to the scientific community–Taken into consideration the regulatory background, and the fact that no intellectual property needs to be protected, it is not acceptable if raw data are not made available to other scientists or competent authorities for reanalysis; and (vi) each study must be performed in a way such that it can be reproduced by others. The use of commercially available laboratory animals is preferred. If, however, in-house strains are used, they should be made available to scientists wishing to reproduce the study.

## How can differences between industry-sponsored and publicly sponsored studies be explained?

There is strong controversy on the weight given to studies that were performed under GLP and according to standard Organisation for Economic Co-operation and Development (OECD) protocols (mostly industry sponsored due to the requirement for GLP and guideline studies for regulatory submission) and exploratory studies (mostly sponsored by public funding). Some scientists (e.g., Myers et al., 2009) claim that “funding by chemical corporations accounts for most other studies that conclude low doses of BPA are safe,” whereas the majority of publicly funded studies show that low BPA doses cause harm (http://endocrinedisruptors.missouri.edu/vom-saal/vomsaal.html) and that studies funded by industry “used insensitive, out-of-date protocols and assays that are incapable of finding many of the adverse effects.” They conclude that these studies are flawed (Myers et al., 2009). However, this way of interpreting differences between guideline-compliant and exploratory research, mainly performed at universities and research institutes with public funding, is naive. It ignores the basically different conditions, goals, and strategies of both types of research. Explorative research at universities is often performed to discover new mechanisms or to analyze how a compound interferes with mechanisms that represent cutting-edge topics of current academic research. Animal numbers usually need to be low, as technical conditions and resources do not allow the treatment, observation, and analysis of high numbers of animals. Statistical evaluation of explorative studies is usually performed by tests accepting differences between compound exposed samples and controls as significant if a *p* value smaller than .05 is obtained without applying an appropriate adjustment for multiple testing. Hence, the findings can only be interpreted as exploratory results and need at least one further study confirming the findings. A *p* value of .05 means that for 100 endpoints in a study where a compound has no influence, 5 endpoints result in (false-)positive results. Considering that many more than 1000 explorative studies, with each study examining multiple endpoints, have been published, we have to expect a relatively high number of “false-positive” findings. Importantly, they are “false positive” only for statistical reasons, not because of false claims or flaws. It is also important to consider that it is much easier to publish positive than negative results of explorative studies in high-ranking journals. This leads to the well-known phenomenon of publication bias. Therefore, it is not helpful to count how many academic studies are positive versus negative and to decide by majority vote whether a health hazard has to be expected or not. An important aspect is also that explorative studies may identify a chemical-induced biological event, but this event may not translate into an adverse health effect. The long-term low-dose safety studies on BPA demonstrate this.

The conditions and the purpose of guideline-compliant regulatory studies are completely different from those of academic studies (Tyl, 2009a, 2009b). Guideline-compliant toxicity testing aims to identify specific and validated endpoints associated with adverse health effects in a well-characterized animal model. The study design and tests to be performed must be documented prior to the study. High numbers of animals and adequate statistical procedures have to be applied. Guideline-compliant toxicity studies also determine a wide range of parameters (hematology, clinical chemistry, etc.) and include full histopathological assessment of all organs considered relevant. Usually, a battery of such guideline-compliant and quality-controlled tests is used in risk assessment in order to analyze a sufficient number of different end-points. Under conditions of guideline-compliant tests, the probability of false-positive results is much lower, particularly because of the higher number of animals, a priori defined study design, and highly sophiosticated statistics.

Usually, there is a fruitful interplay between explorative (university) studies and guideline-compliant (industry or contract research organization) studies. In principle, it is possible that novel effects are detected in explorative studies that are not captured by guideline-compliant studies. If such observations are made, the reproducibility and relevance with respect to adverse health effects should be investigated. If confirmed, observations from explorative studies should indeed be used for risk assessment. There are techniques initially used in explorative studies that were later validated and included into toxicity testing guidelines. An example is the use of primary hepatocytes for drug metabolism studies that, after initial use in explorative studies, has subsequently been approved by the United States Food and Drug Administration (US FDA) (Hewitt et al., 2007). Considering the different condition and purpose as well as the fruitful interactions, it is not adequate to play off explorative against guideline-compliant studies just because the percentages of positive results differ between both study types.

### Toxicokinetics

#### Humans

BPA is well absorbed by the oral route. Völkel et al. reported urinary recovery in human volunteers of 97% of the dose in males and 84% in females after oral administration (2002, 2005), indicating extensive absorption of orally administered BPA (key findings summarized in Tables 4 and 5). It should be underscored that Völkel et al. (2002, 2005, 2008) performed their studies by dosing deuterated BPA in order to differentiate between BPA present due to contamination and BPA resulting from dosing. Recently, the study by Völkel has been criticized by Vandenberg et al. (2010a, 2010b) who deemed it to be of limited validity. Therefore, we compiled all critical comments of these authors and assessed their relevance (Table 6). We came to the conclusion that the criticism by Vandenberg et al. (2010a, 2010b) is not justified and that the pharmacokinetic studies by Völkel et al. (2002, 2005), the results of which are consistent with those of other toxicokinetics studies with BPA in nonhuman primates (Table 5), are useful for describing BPA pharmacokinetics in humans.

**Table 4:** Key conclusions from the toxicokinetics study of Völkel et al. (2002) in human subjects using deuterated BPA [d(16)-BPA]

• 5 mg of d(16)-BPA (60–80 µg/kg/bw) were orally administered to human volunteers; blood and urine sampling was performed at predetermined time points.
• Maximum blood concentrations were measured approximately 80 minutes after administration.
• The half-life of d(16)-BPA was less than 6 hours.
• The administered doses were completely recovered in urine as d(16)-BPA-glucuronide.

*Note*. Similar data were obtained by Doerge et al. (2010b), Kurebayashi et al. (2002), and Tominaga et al. (2006) in non-human primates.

**Table 5:** Kinetic parameters of bisphenol A in blood at comparable single oral doses in primates and rodents

Species	Dose (µg/kg bw)	C_max_ (nmol/L)	*t*_max_ (h)	*t*_1/2_ (h)	AUC (nmol × h × L^−1^	Reference
Rhesus monkeys	100	590 (BPA conjugates)	0.5 (total BPA)	3.5 (total BPA)	920 (BPA total)	Doerge et al., 2010b
				0.39 (free BPA)	1.5 (free BPA)	
		0.84 (free BPA)				
Cynomolgus monkeys	100	456 (total BPA)	1.0 (total BPB)	9.6 (total BPA)	1162 (BPA-glucuronide)	Kurebayashi et al., 2002
Human subjects	60–80	820 (BPA conjugates) <10 (free BPA)	1.35 (total BPA)	5.3 (terminal) 1.5 (initial)	2792 (BPA-glucuronide)*	Völkel et al., 2002
Sprague-Dawley rats, female	100	73 (total BPA)	2 (both free and total)	4.6 (total BPA)	680 (total BPA) 2.6 (free BPA)	Doerge et al., 2010a
		0.39 (free BPA)		3.0 (free BPA)		

* Calculated based on a clearance of 7.8 L/h and an oral bioavailability of 99%.

**Table 6:** Data on the fate of BPA in humans have been published by Völkel et al., 2002, 2005*

Criticism	Assessment of the commission
1.“Several inconsistencies are present in this report and therefore raise questions about its reliability (Völkel et al., 2002). For instance, the authors report two different values when maximal plasma concentrations (C_max_) were achieved (1.35 hours versus 4 hours).”	The authors definitely do not report two different time points for maximal plasma concentrations. In fact, they wrote, “The results from this study show that maximal plasma concentrations of d_16_-bisphenol A glucuronide were reached approximately 80 min after oral administration” (Völkel et al., 2002; p. 1285). The blood concentrations of d(16)-BPA-glucuronide are summarized in Figure 6 (Völkel et al., 2002; p. 1285) and show only one peak approximately 80 minutes after administration of BPA and not two peaks as claimed by Vandenberg et al. (2010a, 2010b). As clearly stated in the publication, two separate studies were performed giving identical doses. The first study was intended to provide mass balance/recovery and observe blood levels over a longer time period. Urinary and blood concentrations were determined after oral administration of BPA. In this first experiment, blood concentrations were measured 4, 8, 12, 16, 24, and 32 hours after BPA administration and the highest concentration of BPA-glucuronide was observed after the earliest examined period of 4 hours (Figure 5B in Völkel et al., 2002). In the second study blood levels of BPA-glucuronide were analyzed in shorter intervals of 0, 51, 81, 111, 141, 201, 261, 321, and 381 minutes (Figure 6 in Völkel et al., 2002) to determine initial kinetics. This resulted in a kinetic profile with a single peak after approximately 80 minutes. In fact, extrapolation of the half-life from the first study exactly predicts the levels at the sampling points in the second study and blood concentrations of BPA-glucuronide at overlapping sampling points from the two studies are essentially identical. It should be considered that the first experiment was needed to assess recovery and kinetics in blood at later time points, which cannot be studied with the design required for assessment of distribution and initial elimination from blood, because the latter requires short sampling intervals and thus blood sampling by a venous port. It should also be noted that the authors (Völkel et al., 2002) never mentioned “two different values for the time when C_max_was achieved” in their paper. In conclusion, this criticism of Vandenberg et al. (2010a, 2010b) is not justified.
2.“Additionally, the BPA-glucuronide levels reported in blood are higher than the total BPA concentrations measured in the same individuals.”	The blood concentrations of d(16)-BPA-glucuronide and total d(16)-BPA after glucuronidase treatment are very similar with overlapping error bars as shown in Figure 6 (Völkel et al., 2002). There is no significant difference. Vandenberg et al. may have misinterpreted Figure 6 because the peak values of d(16)-BPA-glucuronide determined by LC-MS/MS are slightly above total d(16)-BPA determined after glucuronidase treatment. However, the difference is in the range of the experimental error. In conclusion, Figure 6 of Völkel et al. (2002) clearly shows that concentrations of total and glucuronidated BPA in blood are very similar after oral administration of BPA.
3.“Finally, the authors indicate that they measured BPA metabolism in 3 women, a group of 3 men, and then in a separate group of 4 men, yet the groups of male volunteers clearly overlap, making the data compiled from combining these two groups questionable.”	The characteristics (gender, age, height, body weight) of the individuals who joined the pharmacokinetic study are given in Table 1 (Völkel et al., 2002, p. 1282), together with identification numbers. Urinary excretion was analyzed in individuals A, B, C, E, F, and G, as described in Materials and Methods. First, since a range-finding study was not carried out for all male individuals, data from four subjects had to be handled separately. Second, BPA-glucuronide concentrations in blood of these individuals were followed over relatively long periods (4, 8, 12, 16, 24, and 32 hours) after administration of BPA (Figure 5) in the first experiment. In the second experiment, the kinetics in blood were determined using shorter intervals (Figure 6). This second experiment was performed with individuals G, M, N, and O (Figure 6). Therefore, only one individual (G) took part in both studies and the determined blood concentrations are highly consistent between the two studies (see above). The commission sees no reason why this constellation should compromise the results, particularly since the study design was clearly described.
4.Vandenberg et al. (2010a, 2010b) criticized that “there was alack of acknowledgement of the likelihood of different toxicokinetics when BPA exposure is continuous compared to a single administration. Ginsberg and Rice suggested that results from the Völkel et al. study were more consistent with delayed excretion from long-term internal storage or cycling between conjugation and deconjugation (Ginsberg and Rice, 2009).”	First, it should be emphasised that the aim of the study of Völkel et al. (2002) was to analyze toxicokinetics of BPA after administration of a single dose. Therefore, this argument cannot be used to criticize the study. Second, basic pharmacokinetic calculations permit the prediction of blood concentrations after repeated doses once the half-life and clearance of a compound are known. The relevant parameters for such calculations are available from Völkel et al. (2002). It should also be kept in mind that the argument of Ginsberg and Rice (2009) is highly questionable, since it is based only on a higher blood concentration at only one time point (24 hours) in one gender where error bars clearly overlap and the differences are not statistically significant. In addition, none of the available other primate studies give any evidence for cycling of BPA. Long-term internal storage or cycling is also very unlikely, since most of the administered compound is recovered in the urine within 24 hours (Völkel et al., 2002).
5.Vandenberg et al. also criticized that “the potential for BPA to have actions at low levels (in the ng/ml range) was not considered” in the Völkel study.	Again, this is not the aim of a toxicokinetics study. The commission has the impression that Vandenberg et al. (2010a, 2010b) confuse the goals of a toxicokinetics study and risk assessment. We comment on this under “criticism 12.” The relevance of low doses of BPA has been discussed insection “Do oral low BPA doses below 5 mg/kg bw/day cause adverse health effects in laboratory animals?” of this article. It is important to note that recent pharmacokinetic modeling studies have shown that the highest reported oral exposures for BPA in the general population would result in blood concentrations much lower than concentrations claimed to cause some effects in vitro (Mielke and Gundert-Remy, 2009).
6.“Third, this toxicokinetics study was designed to assess the metabolism of BPA following oral exposure because until very recently (Stahlhut et al., 2009), it was assumed that most if not all BPA exposure in humans was occurring via the oral route. All sources of BPA have not been identified, so non-oral exposures cannot be discounted.”	This is not a valid argument against the quality of the study of Völkel et al. (2002) because this study aimed at analyzing the pharmacokinetics after oral administration of BPA. We have discussed the question of oral versus non-oral exposure in section “Specific exposure conditions” of this article. Briefly, it should be kept in mind that BPA is quantitatively excreted via the urine. Techniques to estimate overall uptake of BPA from urinary concentrations are generally accepted (and discussed in Mielke and Gundert-Remy, 2009).
7.“Finally, the possibility of differences in toxicokinetics under different physiologic paradigms was overlooked…. The differences between sexes and age groups in urinary levels of BPA found in biomonitoring studies from CDC (Calafat et al., 2005, 2008) raise the possibility that toxicokinetics of chemicals and drugs including BPA are likely to be different in fetuses and neonates compared to adults.”	Analysis of differences between neonates and adults, etc., was not the aim of the study of Völkel etal. (2002). Again, Vandenberg et al. (2010a, 2010b) mixup the goals of a toxicokinetics study and the risk assessment process. For risk assessment, safety factors are used to consider possible differences between neonates and adults. In this context, the finding that a recently published PBPK model predicted the internal exposure in newborns to be approximately 3 times higher than in adults should be considered (Mielke and Gundert-Remy, 2009). The prediction model took into account conjugation by sulfotransferases, which is already active in newborns (Milke and Gundert-Remy, 2009). Moreover, toxicokinetic data from neonatal non-human primates are available (Doerge et al., 2010b).The 2008 Calafat study simply measured urine from neonates in intensive care units and found that urine samples mostly contained conjugates, and the two groups of neonates investigated in this study had major differences in average exposures. The authors suggest that the neonates were exposed to BPA via intravenous medical devices used at one of the sites. The study of Calafat did not examine toxicokinetics of BPA.
8.“BPA kinetics were examined in six individuals administered BPA, although no information was provided about the characteristics of these subjects making it difficult to draw any conclusions from this study (Völkel et al., 2005).”	The authors give detailed characteristics of the subjects participating in the study. In Table 1 (Völkel et al., 2005, page 1749), gender, age, height, and body weight of all six individuals are stated. Further information is given in the Materials and Methods section: “All subjects enlisted in the study had to refrain from alcoholic beverages and medicinal drugs 2 days before and throughout the experiment. Subjects did not abuse alcohol and were nonsmokers. Subjects were healthy, as judged by medical examination and clinical blood chemistry.” Therefore, the statement by Vandenberg et al. (2010a, 2010b) that “no information was provided” is unsubstantiated.
9.“The authors suggested that there were no differences in kinetics between volunteers, yet a closer look shows a wide variation in BPA measurements between individuals (Völkel et al., 2005). In the three men examined, 85 % of the administered BPA dose was recovered in urine after 5 h, mostly as BPA-glucuronide. In the three women examined, 75 % of BPA was recovered as BPA-glucuronide after the same period of time.”	There is no “wide variation in BPA measurements between individuals.” The data (Völkel et al., 2002) show a 93 ± 19 nmol recovery for men and 83 ± 16 for women. Using a second technique analyzing total BPA after enzymatic cleavage ofglucuronides, recovery was 106 ±16 nmol and 92 ±16 nmol, for men and women, respectively. Therefore, the ranges overlap and there is thus no evidence for a wide variation between individuals.
10.“In two of six individuals, unconjugated BPA was detected in the urine at levels of approximately 1 ng/ml. This finding directly contradicts the conclusions reached by the study authors, who suggested that 100 % first-pass metabolism would promptly convert BPA to its conjugated metabolites.”	There is certainly no contradiction and the authors have carefully considered this aspect (Völkel et al., 2002, 2005, 2008). When deuterated d(16)-BPA was used, Völkel et al. did not detect free BPA in the urine. By contrast, when studies were performed with non-deuterated BPA, very low levels of free BPA were detected in some samples. In a further study, the authors even administered both d(16)-BPA and non-deuterated BPA (Völkel et al., 2008). Interestingly, no free d(16)-BPA but free non-deuterated BPA was detected in urine. The authors discussed contamination as a possible reason for the observation that only non-deuterated free BPA was observed. It should also be considered that 1 ng/ml of free BPA represents a very low concentration. A concentration of 1 ng/ml corresponds to a daily intake of approximately 0.025 µg/kg bw, which is 2000-fold below the tolerable daily intake (TDI) of 50 µg/kg bw/day. It should be taken into account that glucuronides of BPA in urine may release free BPA. Therefore, the presence of free BPA in urine is difficult to interpret, particularly if only concentrations close to the LOD are detected.
11.Vandenberg et al. (2010a, 2010b) criticized the fact that LODs of Völkel et al. (2002) were higher than in other studies.	It should be kept in mind that the study (Völkel et al., 2002) is a toxicokinetics study and not a biomonitoring study. In a toxicokinetic study, sensitivity should be sufficient to study the time course of a controlled dose of a chemical for a sufficient period of time. In the case of the study by Völkel et al. (2002), concentrations of d(16)-BPA in urine were above the LOD even 42 hours after administration. The LOD of 10 nM d(16)-BPA in blood is also sufficient, considering that the peak concentrations of the BPA-glucuronide in blood were approximately 800 nM and concentrations could be determined over the time frame covered by the study design. In conclusion, the commission does not see any reason why the toxicokinetics study of Völkel et al. (2002) is compromised because of the sensitivity of the analytical procedure.
12.Vandenberg et al. (2010a, 2010b) criticized the conclusion of Völkel et al. (2002) concluded from their study “that there is no risk from current human exposure levels (Völkel et al., 2002).”	No such statement can be found in the article of Völkel et al. (2002). Moreover, it was designed as a toxicokinetics study and therefore does not make any conclsuion on risk. Again, Vandenberg et al. confuse the goals of a pharmacokinetics study and of risk assessment. Toxicokinetics studies may have important implications for risk assessment; however, risk assessment is not based on toxicokinetics studies per se. Risk assessment is based on NOAELs or benchmark doses from toxicity studies in animals and on exposure assessment. Kinetics can only be used to justify acceptable margins of exposure. It should also be kept in mind that the EFSAhas never based its assessment on the outcome of any of the mentioned BPA kinetics studies but used the species differences in kinetics to conclude that the margin of exposure of 100 is conservative.

* Aspects of these studies have been criticized (Vandenberg et al., 2010a, 2010b). Here, we assess the relevance of the criticism of Vandenberg et al. (2010a, 2010b).

BPA is metabolized to its glucuronide and sulfate conjugates (Hanioka et al., 2008; Kim et al., 2003; Ye et al., 2005). In humans, glucuronidation was described to be catalyzed by the uridine 5′-diphospho (UDP)-glucuronosyltransferase UGT2B15 (Hanioka et al., 2008). More recent work was unable to determine whether UGT2B7 or UGT2B15 is the relevant enzyme (Mazur et al., 2010) but showed that intestinal metabolism does not play an important role in humans. Sulfation is mediated most probably by the sulfotransferase isoform SULT1A1, as SULT1A1 preferentially conjugates phenols (Campbell et al., 1987a, 1987b). In addition, among an array of bacterially expressed SULT isoforms, SULT1A1 had the highest *k*_cat_/*K*_M_ value for BPA conjugation, indicating that this SULT isoform has a relevant contribution to the conjugation of BPA in vivo (Nishiyama et al., 2002). In individuals unintentionally exposed to BPA, glucuronides account for 85% and sulfates for 15% of the oral dose in urine (Ye et al., 2005), whereas sulfates were not identified as metabolites of BPA in experimentally exposed humans (Völkel et al., 2002). Similarly, in a Korean study, the average excretion of the glucuronide in adult men and women was determined to be 80% and 40%, respectively, whereas the proportion of sulfate ester excretion was higher in women than in men (about 40% and 20%, respectively) (Kim et al., 2003). From in vitro data Kurebayashi et al. (2010) calculated that 92% of hepatic clearance is due to glucuronidation and 8% due to sulfation. In studies with experimental oral exposure of 5 mg deuterated BPA, no parent compound but BPA-glucuronide was quantified in plasma and urine. In some biomonitoring urine samples, only small amounts of unchanged BPA were found the amount ranging between a few percent up to 9.5% of the total amount recovered in urine (Dekant and Völkel, 2008; Völkel et al., 2008; Ye et al., 2005). In the study of Völkel et al. (2008), using the identical analytical procedure and equipment as for biomonitoring samples, no deuterated BPA was detected in the urine of a male human subject who ingested deuterated BPA (dose, 60ng/kg bw), whereas trace amounts of non-deuterated BPA were found in these urine samples. The authors attributed this finding to BPA contamination of the urine, e.g., by house dust containing BPA. Others (Doerge et al., 2010a, 2010b; Cao et al., 2010; Sajiki et al., 1999) have also discussed the problem of contamination. In addition to contamination, free BPA detected in urine may be formed from urinary BPA-glucuronide that has undergone enzymatic hydrolysis by autologous or bacterial β-glucuronidase in the urinary bladder (Helander and Dahl, 2005; Ho and Ho, 1985; Paigen and Peterson, 1978; Zenser et al., 1999). Waechter et al. (2007) pointed out that BPA-glucuronide may be unstable in urinary samples under some conditions during storage or analytical work-up steps. Hence, there is overwhelming evidence that unchanged BPA is excreted in the urine in only very low quantities and confounding by artefacts cannot be excluded (Twaddle et al., 2010).

The half-life of BPA can be estimated from urinary excretion data, assuming that the rate-limiting step is metabolic transformation and not urinary excretion of the conjugated metabolites. From the data of Tsukioka et al. (2004), a half-life of 1.5 hours can be derived in humans. A similar half-life, namely 2.28 hours, has been calculated by Shin et al. (2004) in their physiologically based biokinetic (PBBK) model, whereas Cho et al. (2002) calculated an even shorter half-life of 0.73 hours. Using PBBK modeling, Shin et al. (2004) calculated a volume of distribution at steady state of 1.94 L/kg bw and Cho et al. (2002) calculated it to be 1.71 L/kg bw, with corresponding clearances of 26.6 ml/min/kg bw and 29.0 ml/min/kg bw in humans. Using their clearance value, Shin et al. (2004) calculated that a serum concentration of BPA of 1.49 ng/ml (measured by Takeuchi and Tsutsumi, 2002) corresponds to a daily dose of 100 mg BPA, which is more than 2 orders of magnitude higher than the highest exposure of 0.9 µg/kg/day taken from biomonitoring data of Calafat et al. (Calafat et al., 2005, 2008). Hence, the measured concentrations of Takeuchi and Tsutsumi (2002) using an unreliable enzyme-linked immunosorbent assay (ELISA) (see below in “How can biomonitoring support risk evaluation?”) are highly implausible.

#### Non-human primates

Following intravenous (i.v.) administration of BPA (^13^C_12_-BPA stable isotope–labeled substance to avoid background contamination) to adult monkeys, rapid elimination with a half-life of 3.6 hours was observed (Doerge et al., 2010b). Five minutes after administration, more than 70% of circulating BPA was conjugated, suggesting a rapid metabolism. In contrast to rats (Doerge et al., 2010a), no enterohepatic recirculation was observed in monkeys. After oral administration (100 µg BPA/kg bw), absorption of BPA was nearly complete (Doerge et al., 2010b). The concentrations of free BPA in serum of adult monkeys were very low (<1 nM) and absolute bioavailability of BPA, based on the relation of the areas under the plasma concentration–time curve (AUC_oral_/AUC_i.v._), was 0.2%, indicating a high first-pass effect. The mean serum concentration–time profile for total BPA in rhesus monkeys administered an oral dose of 100 µg/kg bw was similar to that of human volunteers administered a dose of 50–90 µg/kg bw BPA (Völkel et al., 2002). The pharmacokinetic parameters of Doerge et al. (2010) are in fair agreement with those previously reported for aglycone and conjugated BPA in adult male and female cynomolgus monkeys (Kurebayashi et al., 2002; Tominaga et al., 2006). Similar results were recently reported by Taylor et al. (2010) in monkeys, confirming the findings of the other authors.

#### Rats

The most recent study using stable isotope–labeled substance showed a half-life of 0.66 hours following i.v. administration. In this species, more than 50% of circulating BPA was already conjugated at the earliest analyzed time point of 5 minutes, demonstrating a high metabolic turnover. The plasma concentration–time profiles exhibited a second peak in the concentration of total BPA, which points to an enterohepatic recirculation after biliary excretion, as has been previously described by other authors (Kurebayashi et al., 2003; Upmeier et al., 2000; Pottenger et al., 2000). The kinetic paramerets (AUC, elimination half-time, clearance, and volume of distribution) reported by Doerge et al. (2010a) in female rats were comparable to the results of Yoo et al. (2000, 2001) for male Sprague-Dawley rats. A high first-pass effect can be assumed because peak concentrations of total BPA after oral administration contained much lower percentages of unchanged parent compound than observed after i.v. injections (Doerge et al., 2010a). The absolute oral bioavailability of BPA was reported to be 2.8%. This low bioavailability is similar to that reported by Yoo et al. (2001) of 5.3% in adult male Sprague-Dawley rats.

#### Mice

Taylor et al. (2010) published data on aglycone and conjugated BPA in female CD-1 mice demonstrating linear kinetics over a broad range of doses (2 µg/kg to 100,000 µg/kg), a short half-life (∼4 hours), and no accumulation after repeated dosing. In the first serum sample (0.5 hours after dosing) aglycone BPA was ∼1% of the total (aglycone plus conjugated BPA), indicating a high first pass. As in rats, the plasma concentration–time profiles exhibited a second peak in the concentration of total BPA, which points to an enterohepatic recirculation.

## Importance of the exposure route

Exposure of the general population to BPA occurs mostly via food and beverages that have been in contact with polycarbonate plastic. As oral BPA undergoes extensive presystemic elimination whereby glucuronidation accounts to more than 90% (see above), the activity of the metabolites is important to know for risk asessment. As shown by several authors, BPA-monoglucuronide is no longer active as an estrogen (Matthews et al., 2001; Snyder et al., 2000). Since most of human BPA exposure of humans occurs via ingestion (EU, 2003, 2008; Geens et al., 2010; Wilson et al., 2007), laboratory animal studies using the oral route are the most relevant for human risk assessment.

Many of the studies showing adverse effects at low doses of BPA used subcutaneous injections. In others BPA was injected into discrete regions or delivered by osmotic pumps. Unless blood and/or tissue concentrations are monitored to compare to systemic/internal BPA concentrations in humans, the results of such studies are not appropriate for risk assessment purposes. This has usually not been carried out in studies using injections or after implantation of pumps.

Plausible explanations for the effects observed following non-oral administration of BPA are the lack of first-pass metabolism and the slow release of BPA from the oil suspensions injected. Since the administration route in animal tests for human risk assessment should be the same route as human exposure, we see no reasonable argument why administration routes other than oral should be tested. The exception might be exposure by the dermal route. Deviation from testing animals by the oral route is only justified if laboratory animals show a much higher first-pass detoxification than humans. However, as we have shown above in “Toxicokinetics,” this is not the case for BPA.

## Can rodents be used to extrapolate to the human situation with respect to estrogenic activity?

The current TDI for BPA is based on NOAELs derived from studies using rats and mice. Therefore, it is important to know if these rodent species are similarly susceptible to BPA as humans or—critically—whether humans are much more sensitive. An important aspect to consider in this context is the endogenous production of the estrogen 17β-estradiol (E2) in diverse species. A comparison of plasma levels of E2 (taking into account different phases of the reproductive cycle) across mammalian species revealed that mouse, rat, and dog regulate their normal cycle at comparatively low levels of estrogen, whereas the estrogen levels in monkey and human during particular phases of the cycle are 1 to 2 orders of magnitude higher (Günzel et al., 1989). Accordingly, it is plausible that higher exposures to exogenous estrogens will be required to provoke changes in the endocrine regulation of the human organism compared to certain animal species (if other factors such as the pharmacokinetics are similar—see below). Furthermore, across species, there is a pronounced variability in the number of estrogen receptors, even in the same organ; and the affinity of a certain xenobiotic to these receptors may also vary. Therefore, Günzel et al. (1989) concluded that even when there are effects that are clearly mediated via hormone receptors, a direct, quantitative extrapolation from experimental animals to humans is not justified.

A second critical question is whether there are major pharmacodynamic interspecies differences in susceptibility to estrogens. Available data support the conclusion that rats exhibit a similar sensitivity to EE2 compared to humans, or are slightly more sensitive. In addition, the question of possible insensitive rat strains has been intensively addressed (Gray et al., 2010; Health Canada, 2008; Chapin et al., 2008; NTP, 2008). Several experts came to the conclusion that no single rat strain is highly sensitive or resistant to estrogens. Finally, it should be considered that in current drug development, the pharmacological activity of new hormonal drug candidates is still characterized successfully in rodent species before entering into clinical studies in humans. To our knowledge, results of such experiments have not revealed any “low-dose phenomena” as they have been claimed to occur with BPA.

The misunderstanding of the “estrogen-insensitive rat” came from a letter (vom Saal, 2010) asserting that doses of EE2 (of less than 0.5 µg/kg bw/day) included in oral contraceptives did not cause effects in the rat study of Ryan et al. (2010a). It should be noted, however, that such comparisons on the basis of dose alone are misleading. There are notable differences in the oral bioavailability and, hence, systemic availability of EE2 in rats (approximately 3%) and humans (approximately 45%; Kuhnz et al., 1999). Accordingly, a comparison of sensitivity should incorporate a correction factor accounting for differences in the systemic exposure (i.e., area under the curve [AUC] of plasma concentration over time) as a basis, rather than simply a comparison of the external dose based on body weight; it should also refer to the same endpoint of pharmacological activity. This leads to the critical question of interspecies differences in BPA pharmacokinetics. It is well known that humans and monkeys excrete the BPA-glucuronide predominantly via the urine (Kurebayashi et al., 2002; Völkel et al., 2002). In contrast, rats excrete BPA-glucuronide predominantly via the bile into the feces, resulting in enterohepatic circulation (Inoue et al., 2001; Kurebayashi et al., 2003; Upmeier et al., 2000). In addition, the glucuronidation rate of BPA is higher in liver microsomes obtained from rats compared to humans (Elsby et al., 2001a, 2001b). Considering these interspecies differences—enterohepatic circulation in rodents but not in primates and higher glucuronidation rates for rats compared to humans—interspecies extrapolation from the rodent to the primate or human situation is complex. An overview of interspecies differences in BPA kinetics is given in Table 5. The AUC for BPA-glucuronide after single oral doses of BPA seems to be higher in humans compared to cynomolgus and rhesus monkeys as well as rats. On the other hand, higher exposures to exogenous estrogens may be required to provoke changes in the endocrine regulation in humans or monkeys compared to rats, because rats regulate their normal cycle at lower levels of estrogen, as explained above (Günzel et al., 1989). Therefore, it can be expected that the extrapolation factor of 10 for interspecies differences to obtain the current TDI of 50 µg/kg bw/day is conservative.

## Are there susceptible subpopulations?

The risk assessment of a chemical includes consideration of susceptible subpopulations, which require a specific risk assessment. Risk is determined by hazard and by exposure to the chemical. The hazard is expressed in quantitative terms by the NOAEL, which is adjusted by an assessment factor for interspecies differences and intraspecies/intersubject variability. The generally used default factor to account for the intraspecies variability is 10, which is subdivided into a factor of 3.3 for toxicokinetic and an additional factor of 3.3 for toxicodynamic variability (WHO, 2005). A factor higher than 10 may be necessary to cover a higher variability given in special subpopulations. This can be due to particular toxicokinetic features (mainly because of lower metabolism and/or excretion), or due to particular toxicodynamic features (concentration-response relationship shifted to a lower concentration range at which effects are elicited). Lower metabolism and/or excretion, as well as a shifted concentration-response relationship, may be present in a specific subpopulation and cause concern even if the subpopulation is exposed at the same exposure level as the general population. Conversely, a subpopulation with normal toxicokinetic and toxicodynamic patterns may be at risk because their exposure is higher than the worst-case exposure scenario calculated for the general population.

The following section discusses the toxicokinetics and toxicodynamics of BPA with the aim of evaluating whether there may be defined subpopulations at risk. Exposure considerations are also discussed (see “How can biomonitoring support risk evaluation?”), with the exception of the special situation in neonates in intensive care units and the situation in newborns and babies fed using polycarbonate bottles.

### Toxicokinetics in children

One type of subpopulation at higher risk than the “normal” population is defined by the feature that at the same external exposure their internal body burden, expressed as concentration in blood/plasma, is higher than the internalbodyburdenofthe “normal” population. A higher internal body burden might be due to increased absorption or decreased elimination, both of which would lead to an increase in AUC. Because absorption of BPA is nearly 100%, increased absorption due to factors such as age or disease is not a consideration for BPA. Lower metabolic activity would be the key underlying cause for a decrease in elimination as BPA undergoes extensive conjugation via glucuronidation and sulfation. In newborns and infants up to 6 months, glucuronidation activity is known to be reduced, whereas older children have similar activities to adults (Allegaert et al., 2008, Edginton et al., 2006, Gow et al., 2001; Miyagi and Collier, 2007; Zaya et al., 2006). Hanioka et al. (2008) demonstrated that UGT2B15 is one of the enzymes responsible for glucuronidation of BPA in microsomes from adult humans. Experimental data on UGT2B15 in human development have not been reported so far. However, data are available for UGT2B7, which belongs to the same UGT2B subfamily. The data indicate that the glucuronidation activity of UGT2B7 is 5% of the adult level in newborns, increasing to 30% after 3 months, 80% after 6 months, and 100% at the age of 1 year. This information can be used with some confidence to describe the age-dependent pattern of UGT2B15. It is conceivable that at a given external exposure the internal body burden is higher in children (up to 12 months) compared to adults because of reduced metabolic capacity. BPA plasma concentrations in newborns and infants were predicted by two groups using age-specific toxicokinetic models, which implemented the lower metabolic activity via glucuronidation. Using a model with elimination by glucuronidation as the only pathway, Edginton and Ritter (2009) simulated plasma concentrations in the newborn, who at a given external exposure were 11-fold higher compared to concentrations in adults. They, however, did not take into consideration that SULT1A1 mediates sulfation of BPA and that it is already expressed at high levels, even in intrauterine life. Likewise, SULT1E1 and SULT2A1, which are also capable of BPA sulfation, have also been detected and investigated in fetal tissues (Coughtrie, 2002; Gamage et al., 2006; Pacifici and Marchi, 1993; Duanmu et al., 2006; Miki et al., 2002; Stanley et al., 2005). In a second modeling approach, Mielke and Gundert-Remy (2009) implemented both metabolic pathways—glucuronidation (85% of the excretion in adults) and sulfation (15% excretion in adults). They modified glucuronidation in an age-dependent manner, with 5% of the adult value for glucuronidation in the newborn. In the adult, they predicted similar plasma concentrations to those measured by Edginton and Ritter (2009), whereas the predicted plasma concentrations were only 3-fold higher in newborns than in adults and 1.6-fold higher in 3-month-olds than in adults. The result is due to the fact that in subjects with reduced glucuronidation, a greater proportion of BPA is metabolized to the sulfate metabolite. However, as SULT1A1 has a lower intrinsic metabolic clearance compared with UGT2B15, the concentration of BPA is increased (Kurebayshi et al., 2010). Hence, by definition, newborns and infants up to 3 months would qualify as a subpopulation at risk. In addition to the lower glucuronidation activity, it has to be taken into consideration that SULT1A1 is polymorphically expressed in humans. Dependent on the ethnicity, the prevalences of the wild-type allele *1 and the less active alleles *2 and *3 were reported to be 50–90%, 10–30% and 0–3%, the latter being active only in African Americans in a prevalence of up to 20% (Coughtrie 2002). The functional consequences of this known polymorphism towards the metabolism of BPA are uncertain at this time, as 15% enzyme activity versus 50% enzyme activity in blood platelets have been reported versus no impaired functionality in the recombinant enzyme at all (Coughtrie, 2002). For adults, these observations are of no great quantitative importance, as only 15% of the BPA elimination is via this pathway. For newborns and infants, however, where sulfation might be an important pathway, according to the modeling results of Mielke and Gundert-Remy (2009), a reduced enzyme activity would have consequences on the blood concentration of free BPA. If the polymorphism is functionally relevant for BPA metabolism, then the model without the sulfation pathway used by Edginton and Ritter (2009) would describe a worst-case scenario, namely a totally non-functional sulfation pathway. To which extent this scenario is realistic remains open.

In conclusion, in the special subpopulation of newborns and babies up to 6 months, metabolism is impaired and intraspecies variability is greater than the default factor of 3.3. Two considerations play a role for the phar-macokinetic intraspecies variability: first, the fact that the value of 5% glucuronidation activity in newborns describes the median value, which means that there might be newborns and specifically premature infants with a lower than 5% glucuronidation activity. Second, there is the possibility of an impaired sulfation pathway in newborns homozygous for SULT1A1*2 or SULT1A*3, but only in a small fraction of the population. From these facts, it follows that the default factor of 3.3, which is used to account for the toxicokinetic variability in the general population, seems to be large enough to cover the variability in the newborn population.

#### Toxicokinetics in other groups

Pregnant women have been stated to have a generally impaired metabolism of xenobiotics. This is not true; moreover, and specifically for glucuronidation, there are data showing slightly elevated activity compared to non-pregnant women (Anderson, 2005; Hodge and Tracy, 2007). Often, pregnant women are referred to as being at risk, whereas it is meant that the embryo/fetus would be exposed and at a specific risk. When assessing the risk of in utero exposure, the exposure of the fetus depends on maternal blood concentrations. Maternal metabolism is the mechanism by which most xenobiotics, including BPA, are eliminated. Because “accumulation” of BPA in the fetus does not occur and the human placenta does not metabolize BPA, only a very limited amount of the compound gains access to the fetus, which has been shown by ex vivo perfusion of the human placenta (Balakrishnan et al., 2010). As SULT1A1 activity is present from the 26th week of life, fetal metabolism contributes to a certain extent to the overall elimination of BPA (see below) (Pacifici and Marchi, 1993; Duanmu et al., 2006). The elderly have also been stated to exhibit slower metabolism, which is true only to a limited extent concerning phase 1 reactions (Butler and Beck, 2008; He et al., 2006) but not for phase 2 reactions (Court, 2010).

In conclusion, the fetus is not at risk during the prenatal phase, because it is protected by the maternal metabolism. The relevance of BPA exposure via baby bottles for this subpopulation is discussed below. There is no indication that the elderly or pregnant women are at risk, since their metabolic capacity is not impaired.

### Tissue deconjugation of BPA-glucuronide and BPA-sulfate

Ginsberg and Rice (2009) opened up the discussion that tissue BPA concentrations might be higher than calculated due to deconjugation of BPA-glucuronide and BPA-sulfate in tissues. Although there is no doubt on the presence of the deconjugation enzymes β-glucuronidase and sulfatase in several tissues, it should be emphasized that for risk assessment, quantification of reactions and the chemical species present in equilibrium are indispensable. Recently, experimental data on deconjugation of BPA-glucuronide have been published, which allows quantification of this process in the rat fetus (Nishikawa et al., 2010). According to this publication, uterine (maternal) exposure to 113 nmol BPA-glucuronide resulted in a fetal exposure of 147 pmol BPA-equivalents (109.26 pmol BPA-glucuronide in the fetus plus 31.35 pmol BPA in the amniotic fluid plus 6.45 pmol BPA in the fetus), corresponding to 0.13% of the given dose. In the further quantification, we assume that BPA is present in the amniotic fluid because it is excreted by the fetal kidneys as BPA-glucuronide and then converted back to BPA. This assumption leads to the conclusion that 6.45 pmol BPA (in the fetus) are formed from of 147 pmol BPA-glucuronide, which accounts for 4.4% of the dose passed through the placental membrane. Since 0.13% of BPA-glucuronide passes the placental membrane and from this, 4.4% is converted back to BPA in the fetus, it can be estimated that only 0.006% of the maternal BPA-glucuronide is converted back to BPA in the fetus. Edginton and Ritter (2009) simulated an average BPA-glucuronide concentration of 0.15 µg/L (375 pM) for a realistic worst-case external exposure of 1 µg/kg bw/day in a human adult. Using the rat data of 0.006% conversion in the fetus, this would mean that due to the exposure with BPA-glucuronide, the fetal BPA concentration would be 0.0225 pM. Balakrishnan et al. (2010) reported that in human placenta perfusion experiments, BPA does cross the placenta. The concentration at the fetal side is 0.9-fold the concentration at the maternal side. Hence, for a dose of 1 µg/kg bw/day and a resulting concentration of 0.003 µg/L (Edginton and Ritter, 2009; Mielke and Gundert-Remy, 2009), fetal BPA exposure via blood is 0.0027 µg/L (0.9 × 0.003 µg/L) (11.8 pM). The BPA concentration is added to the BPA concentration formed by the deglucuronidation of BPA-glucuronide, giving a value of 0.0225 pM at 1 µg/kg bw/day (see above). This estimate reveals that at a maternal exposure of 1 µg/kg bw/day (which represents a highly conservative estimate; see below in “How can biomonitoring support risk evaluation?”), fetal BPA exposure is 11.8 pM, whereby passage of BPA-glucuronide through the placenta and deconjugation contributes to the exposure with an amount of 0.2%. Deconjugation of estrone sulfate by steroid sulfatase (STS) is an important mechanism for the intracellular availability of estrone. Estrogen sulfatase is a microsomal enzyme and is ubiquitously distributed in several mammalian tissues, among which the liver, placenta, and endocrine tissues exhibit relatively high activity (Iwamori, 2005). Tan and Pang (2001) characterized the process in liver cells in vitro by reporting *K*_M_ and *V*_max_ values. Valle et al. (2006) found that the specific enzymatic activity of STS in adipocytes was 118 pmol/10^6^ cells per hour, approximately 50–100 times lower than in the placenta. According to Stowell et al. (2006), BPA-sulfate (BPAS) and -disulfate are substrates for STS. In their in vitro system exposing MCF-7 cells to BPAS, desulfation BPA-disulfate and uptake of BPA were observed. Stowell et al. (2006) concluded that sulfation may increase the estrogenic potential of xenobiotics. They observed increased levels of BPA in their cellular system after incubation with BPA-sulfate, because of intracellular deconjugation to the active form. However, given the fact that sulfation is a minor pathway in infants older than 1 year and adults, with only 15% of a BPA dose being sulfated, even a rapid and complete cellular uptake and deconjugation by STS would increase the available amount by 15%, which is not a dramatic increase. In newborns and infants up to 3 months, conjugation to BPA-sulfate becomes an important metabolic pathway because of the lower glucuronidation activity. Hence, deconjugation by STS might have a relevant impact on the availability of BPA at the cellular level. Data in humans on expression and activity of STS are not available. However, the maturation of sulfatase activity has been investigated in developing rats by using triiodothyronin sulfate (T S) as a substrate (Huang et al., 1996). In hepatic microsomal preparations from fetal rats, desulfation activity was extremely low. There was a non-significant trend of increasing desulfation activity in rats after birth until 1 month of age. Desulfating activity increased between the 1- and 2-month-old groups to reach adult levels at the end of the second month, mainly due to increased enzyme capacity. If the results in the rats are applicable to humans, it could be concluded that due to lacking STS during the first months of life, deconjugation of BPA-sulfate does not occur to a significant extent. Hence, even in the age groups in which conjugation to BPA-sulfate becomes an important metabolic pathway, availability of BPA at the cellular level is not increased due to low expression of STS in this age group.

### Toxicodynamics

Due to its estrogenic property, BPA is expected to have an impact on physiological processes that are influenced by estrogens. To obtain an insight into the possible impact of BPA, it is helpful to compare BPA levels in maternal blood with their levels of estrogens during pregnancy. Increasing gestagen and estrogen concentrations are observed in the course of pregnancy. Whereas 17β-estradiol serum levels in women in the reproductive age vary between 50 pg/ml (menstruation) and 200 pg/ml (follicular development) (0.18 nM and 0.73 nM), they are 3000 pg/ml (11 nM) at 12 weeks and increase to 25,000 pg/ml (92 nM) at week 40 of pregnancy (Salas et al., 2006). When the concentrations of estradiol are compared with the predicted concentration of BPA of 0.003 µg/L (11 pM) at the highest exposure levels in adults (1 µg/kg bw/day), the ratio between estradiol and BPA increases from 60-fold (persistent follicle) to 8000-fold at week 40. Taking into account the much lower estrogenic potency of BPA, it is obvious that BPA does not contribute to a biologically relevant extent to the total estrogen exposure during pregnancy.

## Specific exposure conditions

### Neonatal intensive care unit

Specific exposure conditions to BPA were reported by Calafat et al. (2009) in patients of a neonatal intensive care unit. The mean urinary concentration of BPA-glucuronide in a single urine sample of this population was 30.3 µg/L, with the highest individual measured value was 946 µg/L. Unfortunately, no clinical details on the neonates were reported, so a number of assumptions have to be made for further calculations. Taking 300 ml as the urine volume per day and a neonate body weight of 3 kg (International Commission on Radiological Protection, 2002), this gives a median intake of 3.03 µg/kg (maximum intake of 94.6 µg/kg) and a median BPA steady-state plasma concentration of 0.026 ng/ml (maximum steady state concentration 0.83 ng/ml). An intake of 94.6 µg/kg exceeds the TDI of 50 µg/kg bw/day derived for the adult based on oral rat data. Hence, neonates in intensive care units may have a specifically high exposure to BPA, most probably because of intravenous exposure to products containing polycarbonates. Exposure on a neonatal intensive care unit is not for the whole life and this has to be taken into consideration for risk assessment. However, 20% of the calculated concentrations range above 1 nM (= 0.23 ng/ml). In in vitro studies with human adipocytes, this concentration has been reported to stimulate mouse β-cell insulin production and secretion by activation of the extracellular signal-related protein kinase 1/2 pathway and to inhibit adiponectin release (Vom Saal and Myers, 2008). In conclusion, neonates in intensive care units may be exposed to BPA by the intravenous route in high amounts. Under those conditions, calculated internal BPA concentrations are in the concentration range that elicits effects in in vitro studies.

### Polycarbonate (PC) bottles

There are several studies demonstrating that BPA migrates from PC feeding bottles. Leaching varies among products and experimental conditions such as temperature and duration of the procedure (Brede et al., 2003; Cao and Corriveau, 2008; EFSA, 2006). Migration can increase with repeated use of the bottles because of cleaning procedures. An upper value of migration of 50 µg BPA/L was identified in the EU (2003), whereas in two migration studies conducted under realistic conditions of use, the highest level of BPA migration in used PC bottles was measured to be 22 µg/L (Tan and Mustafa, 2003; Brede et al., 2003). Using the worst-case estimate concentration of 50 µg BPA/L from the EU risk assessment report (infant formulae in used bottles), EFSA (EFSA, 2006) calculated an exposure of 11 µg/kg bw/d BPA for a 3-month infant who was fed with infant formula with a PC bottle. The estimate is conservative and represents a realistic worst-case scenario. Infants up to 3 months belong to a sub-population at higher internal exposure according to their altered toxicokinetics. However, the exposure level of 11 µg/kg bw/day does not exceed the TDI modified by an additional factor of 3 to account for interindividual differences in toxicokinetics (TDI of 50 µg/kg bw/day divided by 3 = 17 µg/kg bw/day). Hence, although the exposure in the age group of 3-month-old infants fed with infant formula with polycarbonate bottles is enhanced compared to breast-fed infants, it does not raise concern.

## How can biomonitoring support risk evaluation?

### Results and interpretation of biomonitoring studies with BPA

Biomonitoring is a direct approach to estimate human exposures to chemicals from environmental and occupational sources. Due to highly sensitive analytical chemistry, biomonitoring has developed into a valuable tool in exposure assessment (Angerer et al., 2007; Boogaard, 2007; Calafat and Needham, 2007, 2009; Needham et al., 2007) However, transforming biomonitoring data to a daily dose requires a detailed understanding of the toxicokinetics of an agent. Moreover, due to the high sensitivity of modern analytical chemistry, detailed quality control and reduction of potential contamination with the analyte from other sources is needed (Calafat and Needham, 2007; Dekant and Völkel, 2008; Hoppin et al., 2006).

Regarding BPA, a large number of biomonitoring studies are available (for overviews see Dekant and Völkel, 2008; Vandenberg et al., 2007, 2010a, 2010b). Concentrations of BPA present in both urine and plasma of the general population are often close to the limits of quantification (LOQs), even using highly sensitive methods, and this, together with the many confounding factors associated with these methods, has raised specific issues in data generation and evaluation that need to be addressed (Calafat and Needham, 2009; Dekant and Völkel, 2008; Markham et al., 2010; Ye et al., 2007):

- Stability of BPA and BPA conjugates in the sample matrix and during sample processing.
- Sample handling and sample work up; for example, avoidance of polycarbonate-containing plastic tools such as tubes or syringes.
- Measures to reliably reduce background of BPA in sample blanks to levels below the LOD.
- Variability of background present in blanks.
- Use of appropriate internal standards, especially stable isotope–labeled derivatives.
- Description of sensitivity and specificity of the applied analytical method.
- Detailed quality control of the analytical method and sample processing including recovery, precision, and reproducibility.

The results of the many biomonitoring studies on BPA have recently been reviewed and will not be reiterated in detail here (Dekant and Völkel, 2008; EFSA, 2006; US FDA Memorandum, 2009a, 2009b; Vandenberg et al., 2010a, 2010b). Moreover, since only urine or blood concentrations of BPA and its metabolites are useful in exposure assessment, the comments concentrate on these two matrices.

In the large number of urine samples analyzed for BPA (> 10,000), most reported concentrations of total BPA were well below 10 µg/L (Bushnik et al., 2010; Calafat et al., 2005, 2008; Lakind and Naiman, 2008, 2010); higher concentrations were present in a very limited number of samples (Garcia-Prieto et al., 2008; Moors et al., 2007). Very high concentrations of BPA and BPA conjugates were only observed in the urine of newborns from one intensive care unit (Calafat et al., 2009) and in a study from China (Mao et al., 2004). The high urinary excretion of BPA conjugates in the newborns are likely to be related to BPA release from medical equipment (see above). Most of the BPA in urine is present in the form of conjugates when separate analysis for free BPA and BPA conjugates was performed. This observation is consistent with results from toxicokinetics studies in both humans and non-human primates with BPA after oral administration (Kurebayashi et al., 2002; Tominaga et al., 2006; Tsukioka et al., 2003, 2004; Uchida et al., 2002; Völkel et al., 2002, 2005, 2008).

Blood concentrations of “free” BPA of up to 22 µg of free and 66.48 µg of “total” BPA/L have been reported in maternal and fetal blood samples at delivery (Padmanabhan et al., 2008; Schönfelder et al., 2002b), whereas many other studies reported much lower concentrations of BPA (usually less than a few micrograms of “total” BPA/L) in blood of the general population. It has been claimed that the high concentrations reported in maternal or fetal blood at delivery suggest high exposures of the general population to BPA from unknown sources, likely through pathways where BPA is not metabolized by an intensive first pass. However, a detailed analysis of the database on reported blood concentrations of BPA, considering the strengths and weaknesses of the analytical methodologies, sampling procedures, background contamination, and biological plausibility based on the toxicokinetics, needs to be performed to draw conclusions.

A part of the database on blood, serum, or plasma concentrations of BPA is based on ELISAs (enzyme-linked immunosorbent assays). Some ELISA-based report concentrations of “free BPA” in the range of a few micrograms per liter. However, ELISA assays have been demonstrated to widely overestimate BPA concentrations actually present and are cross-reactive with BPA-glucuronide and other constituents in blood or plasma (Lee et al., 2008). Moreover, different ELISA kits gave widely differing results with identical samples, and the BPA concentrations indicated by ELISA were inconsistent with BPA concentrations determined by instrumental analytics (Fukata et al., 2006; Tominaga et al., 2006). Therefore, ELISAs are not reliable to quantify the low concentrations of BPA present in blood samples of the general population.

The studies examining BPA in maternal and fetal blood samples also have a number of drawbacks that renders them unsuitable for an assessment of population exposures (Lee et al., 2008; Padmanabhan et al., 2008; Schönfelder et al., 2002b; Vandenberg et al., 2010a, 2010b):

- The extent of medical intervention during delivery is not reported. BPA may be released from medical devices (Calafat et al., 2009). Thus, exposure may have occurred by the intravenous route, therefore representing a specific situation (see above); the measured concentrations of BPA may therefore have no relevance for the general population.
- The publications have major problems regarding methods and reporting. Enzymatic cleavage of BPA conjugates was not carried out, thus only “free” BPA was determined.
- Despite considerable efforts to reduce BPA concentrations in blanks and contrary to statements made in the text, BPA peaks remain in blanks presented in the figures (Schönfelder et al., 2002b) and the variability of the background is not given. Moreover, a number of inconsistencies between the text and the figures regarding calibration were identified (e.g., a limit of detection [LOD] cannot be determined with BPA appearing in the blanks; the peak area of the internal standard varies widely, suggesting low reliability of the reported BPA concentrations).
- In the second paper (Padmanabhan et al., 2008), a very short solvent gradient is applied and pure methanol is used as eluting solvent during most of the separation. Due to the high eluting capacity of methanol, separation efficiency is low. BPA is also eluted from the column long after the gradient is reversed back to initial conditions. This is inconsistent with theory and practice of reversed phase liquid chromatography. Therefore, this methodology cannot be considered as reliable.

A third study (Lee et al., 2008), which reported blood levels of BPA at delivery, determined BPA after hydrolysis by high-performance liquid chromatography with postcolumn fluorescence derivatization (HPLC-FLD). Some of the results were confirmed either by gas chromatography–mass spectrometry (GC/MS) after derivatization or by liquid chromatography–mass spectrometry (LC-MS) (no details given). No information on background induced by the derivatization for GC/MS was given (which may be expected to be high). In addition, HPLC-FLD has the disadvantage of low specificity and separation efficiency.

In blood/serum/plasma samples of the general population, most studies report lower concentrations (usually <1 µg/L) either “total” or “free” BPA; if detected, BPA concentrations were often close to the LODs of the methods applied. Many of these studies did not use specific procedures to reduce contamination and do not give BPA concentrations in blank. Studies with more elaborate quality control and low or absent BPA contamination in blanks often report BPA levels below the LODs or well below 1 µg/L. Interestingly, blank concentrations of free BPA using a sensitive LC/MS-MS system were consistently in the range of 1 ng/L of plasma, identical to the “background” exposure in human blood often cited (Twaddle et al., 2010) and studies with eleborate quality control did not detect BPA in human blood samples with an LOD of 0.3 µg/L (Ye et al., 2009).

### Biomonitoring and exposure assessment

The reliable estimate of systemic doses by biomonitoring may represent an important part of the exposure assessment if a sufficient number of samples have been analyzed. Usually, biomonitoring is more precise than indirect exposure assessments relying on assumption of food consumptions and migration from food contact material or other sources such as breathable air. However, biomonitoring has to transform concentrations measured in biological samples into a daily exposure/dose. For compounds such as BPA that are rapidly metabolized and completely eliminated in the urine, urinary concentrations are much more useful indicators of human exposure compared to blood concentrations. Concentrations of BPA in blood rapidly decline after intake due to the short half-life of “free” BPA. Any exposure assessment based on blood concentrations has to calculate back to consider infrequent food intake, which is very difficult to perform without having very detailed information.

One of the problems of biomonitoring urinary BPA is the variability between different collection periods. However, possible variations in estimates based on urinary concentrations due to urine collection intervals will be averaged due to the availability of a large data set. Concentrations of BPA and its metabolites in spot urine samples may therefore be used to calculate daily BPA exposures based on average 24-hour urine volumes. Using measured urinary concentrations of BPA and considering toxicokinetics, total daily doses of BPA well below 1 µg/kg bw have been derived (Dekant and Völkel, 2008; Miyamoto and Kotake, 2006). The daily exposure of humans to BPA, established by biomonitoring, is thus well below the daily exposure, as derived from indirect estimates of exposure based on food consumption. Such assessments are based on conservative assumptions of food intake and migration data, and, furthermore, integrate the high end of food concentrations of the agent under study. Most regulatory agencies prefer to use such data because they rely on conservative approaches for exposure assessment. However, the US FDA, EFSA, and the Japanese government have evaluated the available biomonitoring studies and concluded that the exposure estimates based on food concentrations and migration of BPA are conservative compared to daily intakes based on biomonitoring.

When translating blood or urine concentrations from biomonitoring to daily intakes, the data should also be consistent with results obtained by other means of exposure assessment. This is the case for BPA:

- The daily dose of BPA derived by biomonitoring of urinary excretion and average daily excretion equates to similarly low mean daily intakes to exposure assessments based on food concentrations of BPA and food consumption patterns, 0.008 µg/kg bw/day (Thomson and Grounds, 2005) or 0.002 µg/ kg bw/day (Miyakawa et al., 2004) for a 60-kg adult. Calculated daily doses of BPA based on measured concentrations in air, dust, and food were between 0.052 and 0.074 µg/kg bw/day in preschool children (Wilson et al., 2007).
- The good agreement of daily BPA intake based on food consumption with intakes based on urinary biomonitoring further support the conclusions that food is the major exposure pathway for BPA. This is consensus among all regulatory authorities performing such exposure assessments.
- Claims that daily human exposure to BPA is much higher from unknown sources by poorly defined path-ways are not substantiated. Concentrations of BPA in house dust are low. BPA is not used in cosmetics and dermal absorption of BPA is limited. The dermal contact area for potentially BPA-containing materials (e.g., credit card slips) is small, dermal penetration of BPA is limited, and the contact time of human skin with such articles is short (EU, 2003). Due to the very low volatility of BPA, exposure via inhalation can also not be considered as a relevant pathway.
- In a recent publication, it was postulated that human exposures to BPA from unknown sources are much higher than previously assumed based on the rapid elimination of “free” BPA from the blood of monkeys after oral administration (Taylor et al., 2010). The authors suggested that the background blood levels of “serum unconjugated BPA” (0.3–4 ng/ml) indicate that human exposures to BPA would have to be at least 0.5 mg/kg bw, which is orders of magnitude above those estimated by a number of regulatory agencies. However, this analysis is highly deficient. Although the authors acknowledge that a significant part of orally administered BPA is excreted with urine in primates, they do not provide any explanation of what happens to the large assumed daily doses as well as completely neglect the consistent urinary biomonitoring data. Exposures to BPA in the range of 0.5 mg/kg bw/day should result in urine concentrations well above 10 mg/L of BPA/BPA conjugates. *Such concentrations (1000-fold higher than maximal concentrations reported) have never been reported in urine biomonitoring with samples from more than* 10,000 human subjects. Therefore, there is no valid basis for the conclusions of exposures to daily doses of BPA in the range of 0.5 mg/kg bw.

In addition to being in agreement with other exposure assessments, biomonitoring data should also be biologically plausible. The high blood concentrations of “free” BPA reported in some biomonitoring studies are inconsistent with predicted blood levels of “free” BPA even when using the high end of estimated intakes based on food concentrations nutritional habits. Very low blood concentrations of “free” BPA are predicted by PBBK modeling based on the toxicokinetics of BPA in humans and primates (Edginton and Ritter, 2009; Mielke and Gundert-Remy, 2009; Teeguarden et al., 2005). Even a simple calculation using pharmacokinetic parameters for BPA support the conclusion that high blood levels of BPA claimed in some studies are not realistic. The volume of distribution of BPA is 2 L/kg (NTP-CERHR, 2007) and the half-life in blood is around 1 hour (Kurebayashi et al., 2002; Taylor et al., 2010; Doerge et al., 2010b; Völkel et al., 2002). Therefore, a blood concentration of 10 µg/L BPA corresponds to an intravenous dose of approximately 3 mg BPA within 1 hour before blood sampling for a 70-kg person. Adjusting to an oral uptake and considering a bioavailability of “free” BPA of 1%, the resulting intake is 300 mg BPA for a 70-kg person, which is inconsistent with all exposure estimates.

## What are the mechanisms of action of BPA? Does the multitude of mechanisms besides estrogen receptor activation make the substance more hazardous?

The estrogenic activity of BPA was first described in 1936 (Dodds and Lawson, 1936). BPA interacts with estrogen receptors ERα and ERβ, with a slightly higher affinity for ERβ. BPA has also been reported to show antiandrogenic activity at approximately 5-fold higher concentrations than those causing estrogenic activity (reviewed in Bondesson et al., 2009). Moreover, low activities have been reported for the pregnane X receptor (PXR), the estrogen receptor–related receptor (ERR), and the thyroid hormone receptor (TR) (Mnif et al., 2007; Okada et al., 2008; Abad et al., 2008; Matsushima et al., 2007; Moriyama et al., 2002; Liu et al., 2010). BPA was also reported to inhibit TH signaling but at higher concentrations than those that interact with estrogen receptors (Fini et al., 2009), and to cause increases in levels of uterine heat shock proteins (hsps), mainly hsp90a and glucose-regulated protein (grp) 94 (Papaconstantinou et al., 2002). The fact that BPA, in addition to its effect on estrogen receptors, also interferes with other receptors has been used to argue that “the risk assessment of endocrine disrupting compounds, such as BPA, is hampered by large scientific uncertainties” (Bondesson et al., 2009). However, does the interaction with other receptors make the compound more hazardous? It should be considered that it is not surprising that a hormonally active chemical is not specific for a single receptor that is known also for several hormonal drugs. The potential to interact with a receptor is not per se indicative of a toxicologically relevant effect. The fact that BPA activates estrogen receptors as well as other receptors fits into a scenario frequently observed for hormonally active chemicals as well as for hormonal drugs interacting with several receptors with different affinities. In addition, it can never be excluded with certainty that further relevant toxic mechanisms have notyet been discovered. However, an important argument is that current risk assessment of BPA is based on a large number of adverse endpoints in a multitude of animal experiments (see above). These in vivo studies capture adverse health effects potentially induced by all receptor interactions, without requiring an a priori knowledge on the involved mechanisms. Of course, it is an advantage if the mechanisms responsible for observed adverse health effects are known because this offers the opportunity to refine risk evaluation, for example by comparing critical mechanisms in rodent and humans in order to identify possible interspecies differences. However, the risk to humans is only underestimated if critical toxic mechanisms are more active in humans compared to the animal species that were used for the toxicity studies. Considering the available studies on BPA, there is no evidence for such a critical (toxic) mechanism that is specific for humans or where human cells or tissues are more sensitive than the respective tissues or cells from animals.

Evaluating the relevance of ERα- and ERβ-mediated toxicities, it should be noted that BPA is about 10,000-fold less potent than estradiol (Gray, 2008). Because of the low levels of human exposure to BPA, it is unlikely that toxicity is mediated via estrogen receptors in humans. It should also be considered that genomic studies in rodents exposed to low doses of BPA did not result in expression of estrogen receptor–dependent genes in rat uterus or fetal rat testis (Ashby and Odum, 2004; Naciff et al., 2005, 2010). Induction of estrogen receptor–dependent genes by BPA was only observed at moderate to high dose levels with no evidence of non-linear dose-response. Evaluating the relevance of the weak estrogenicity of BPA, it should also be considered that humans are exposed to a variety of dietary natural compounds with higher estrogenic activity and at higher doses than BPA (Bolt et al., 2001; Safe 2000, 2004).

### Epidemiological studies in the general population

In a limited number of epidemiological studies BPA exposure data were related to health outcomes. The majority of the studies have a cross-sectional design where a single urinary BPA level is used as exposure estimate. Health outcomes were analyzed mostly by self-reported methods (e.g., questionnaires), and had a long latency period such as for cardiovascular disease (Melzer et al., 2010) or diabetes (Lang et al., 2008; Melzer et al., 2010). The same holds true for cross-sectional studies on semen quality and sperm DNA damage (Meeker et al., 2010a, 2010b), serum testosterone, estradiol, and sex hormone–binding globulin (Galloway et al., 2010). Given the short half-life of BPA and the long latency of the health outcomes addressed, the results of the cross-sectional studies concurrently are hard to interpret. Therefore, the above-mentioned studies report associations that can at best raise hypotheses rather than demonstrate causal relationships. Also the case-control studies on breast cancer with relatively small case numbers (Yang et al., 2009) suffer from the time lag between an actual single urinary excretion of BPA as the exposure estimate and the time of occurrence of the disease. At present, there are no studies confirming the results available.

## Recent governmental responses

Risk management is a decision-making process to select the optimal measures for reducing a risk to an acceptable level. It follows the risk assessment step (optimally incorporating the description of uncertainties in the risk assessment process) and involves consideration of political, social, economic, and engineering factors. Risk perception by society and hence regulatory authorities also reflects the knowledge of toxicology and the culture of the society and may change with time as more information becomes available. The risk management process is iterative, taking into account any new information. Risk management decisions can therefore involve measures to prevent the process producing the risk, measures to reduce or eliminate exposures, and activities to alter perceptions or valuation. The basic options in risk management are setting toxicologically based guidance values or applying a more rigorous precautionary approach, e.g., restricting concentrations/doses to levels achievable by the best available technology.

National bans on BPA in baby bottles, which have been adopted in recent months by some countries, are based on a precautionary approach. There is no scientifically proven increased risk discernable for the age group of infants fed with polycarbonate bottles. Against the background of an ongoing controversy, it is easily understood that the current heterogeneous situation in governmental responses reflects political motivations in the respective countries rather than a scientifically justified systematic risk management. The committee therefore refrains from any scientific comment but tries to present an objective overview on the actual situation.

### European Union

BPA is permitted for use in food contact materials in the European Union, under Commission Directive 2002/72/EC of 6 August 2002 relating to plastic materials and articles intended to come into contact with foodstuffs. In its risk assessment on BPA published in January 2007, the EFSAsetaTDI of 0.05 mg/kg bw/day for BPA. EFSA found that intakes of BPA through food and beverages were well below the TDI, even for infants and children. EFSA's risk assessment of BPA was updated in July 2008 and October 2010 and the TDI (0.05 mg/kg bw/day) was reconfirmed (EFSA, 2008, 2010a, 2010b).

EFSA found that intakes of BPA through food and beverages were well below the TDI, even for infants and children. Typical BPA migration levels from BPA-based food contact materials are <10 µg/kg food and thus are well below the regulatory specific migration level for BPA of 600 µg/kg food (based on the TDI, assuming a person of 60 kg eating 1 kg of food per day, with an additional safety factor of 5). Using conservative migration levels, EFSA concluded in their 2007 opinion that the dietary exposure to BPA from polycarbonate plastic bottles and epoxy resin–coated food and beverage cans is well below the TDI. The updated risk assessment of JunE 2008, as well as EFSA's updated opinion of July 2008, states that food contact materials such as polycarbonate plastic baby bottles, drinking bottles, and epoxy resin–coated food and beverage cans are safe for their intended uses (EFSA, 2008). In its opinion, EFSA examined the safety of BPA-based food contact applications for all age groups, including fetuses and newborns.

EFSA updated its advice on BPA in September 2010. Following a review of recent human toxicity data and animal studies on the toxicity of BPA at low doses, scientists of EFSA's Panel on Food Contact Materials, Enzymes, Flavourings and Processing Aids (CEF) concluded they could not identify any new evidence that would lead them to revise the current TDI for BPA of 0.05 mg/kg bw set by EFSA in its 2006 opinion and reconfirmed in its 2008 opinion (EFSA, 2010a, 2010b). The Panel also stated that the data currently available do not provide convincing evidence of neurobehavioral toxicity as an endpoint of concern for BPA. The present opinion follows the requests of the European Commission to the CEF Panel to evaluate the dietary developmental neurotoxicity study of BPA in rats by Stump et al. (2010) and recent scientific literature (2007 to July 2010) in terms of relevance for the risk assessment of BPA and impact on the current tolerable daily intake (TDI) of 0.05 mg BPA/kg bw/day and to provide advice on the Danish risk assessment underlying the Danish ban of BPA in food contact materials for infants aged 0–3 years.

### Denmark

On the basis of an assessment by the National Food Institute at the Technical University of Denmark (DTU Food) (http://www.food.dtu.dk/Default.aspx?ID = 8590) released 22nd March 2010, the Danish government decided on 26th March 2010 to invoke the principle of precaution and introduce a temporary national ban on BPA in materials in contact with food for children aged 0–3 years (infant feeding bottles, feeding cups, and packaging for baby food). From 1st July 2010, BPA is not allowed in the products covered by the ban.

The overall assessment of DTU Food concluded that the new neurodevelopmental study by Stump et al. (2010) does not shed new light nor change the uncertainties about the impact of small doses of BPA on the development of the nervous system and the behavior of rodents. The conclusion from DTU Food is that this study does not give clear evidence of BPA harming the behavioral end-points examined. However, it raises uncertainties about the impact on learning capacity. In their opinion, the study revealed reduced learning capacity of young male rats at low doses of BPA. According to the opinion of DTU Food, the finding of reduced learning capacity of newborn males may be a sign of low-dose effect of BPA. However, they also discuss that this observation may just be coincidental. The Danish Veterinary and Food Administration adopted DTU Food's opinion. On this basis, the Danish Veterinary and Food Administration concluded that the precautionary principle dictates the introduction of protective measures with respect to children aged 0–3 years until new studies document that low doses of BPA do not have an impact on development of the nervous system or on the behavior of rats.

### France

On 25th March 2010, the French Senate called on the government to suspend the commercialization of BPA-based polycarbonate baby bottles in France, until the French Food Safety Authority (AFSSA) issued conclusions on their ongoing assessments of BPA. A ban on manufacturing, importing, exporting, and selling of baby bottles made of BPA-based products has been approved by the National Assembly on 11th May 2010. The ban was endorsed under the Grenelle II sustainable development initiative. This is a temporary ban until AFSSA develops new methods for evaluating risks linked to BPA in food containers used by children under the age of 3. The French ban came into force on 30th June 2010 (http://www.legifrance.gouv.fr/affichTexte.do;jsessionid=?cidTexte=JORFTEXT000022414734&dateTexte=&oldAction=rechJO&categorieLien=id).

### Switzerland

The Swiss Federal Office of Public Health (FOPH) (February 2009) evaluated the scientific assessments of various international governmental agencies and came to the conclusion that exposure to BPA poses no risk for consumers including neonates and infants.

### Australia and New Zealand

Food Standards Australia New Zealand (FSANZ) has evaluated the safety of BPA and plasticizers in baby bottles and concluded that levels of intake of BPA or plasticizers are very low and do not pose a risk to infant health. In contrast, a voluntary phase out by major retailers began on 1st July 2010. It is the result of discussions between the Australian Government and retailers.

The Australian Government appreciates there has been a level of public concern relating to BPA in baby bottles and, therefore, has worked with retailers to introduce the phase out (http://www.foodstandards.gov.au/scienceandeducation/newsroom/ mediar-eleases/mediareleases2010/governmentannounc-esb4822.cfm).

### Canada

Under the Government of Canada's Chemicals Management Plan (December 2006), BPA was identified as a high-priority substance for assessment of human health risk. Environment Canada and Health Canada considered in their joint Screening Assessment from October 2008 that the neurodevelopmental and behavioral data set of BPA for rodents, though highly uncertain, is suggestive of potential effects at doses of the same order of magnitude to 1–2 orders of magnitude higher than exposures to BPA of the general population. Given that toxicokinetics and metabolism data from experimental animal and limited human studies indicate potential sensitivity to the maternal-fetal unit and infant, and that animal studies suggest a trend towards heightened susceptibility during early stages of development in rodents, it is considered appropriate to apply a precautionary approach when characterizing risk. In conclusion, BPA should be considered as a substance that may be entering the environment in a quantity or concentration or under conditions that constitute or may constitute a danger to human life or health in Canada.

A provisional TDI of 25 µg/kg bw/day was preestablished by Health Canada as a conservatively safe level for BPA presence in food and was confirmed in the 2008 Health Risk Assessment of BPA from Food Packaging Applications (Health Canada, 2009b). The prohibition of polycarbonate baby bottles that contain BPA came into force on 11th March 2010 (Canada Gazette Part II, 31st March 2010), thus prohibiting the advertisement, sale, and import of these products in Canada. Furthermore, the Canadian Government works to develop and implement codes of practice to reduce levels of BPA in infant formula as low as reasonably achievable. The ban was enforced after the market for polycarbonate baby bottles had virtually disappeared. The decision was made in spite of the results of four migration studies carried out by Health Canada and published in 2009. The studies specifically investigated migration from polycarbonate baby bottles, canned soda, canned infant formula, and bottled water, and found no or extremely low migration levels, thus confirming existing data of very low exposure (Health Canada, 2009a, 2009b, 2009c, 2009d).

### USA

The National Toxicology Program Center for the Evaluation of Risks to Human Reproduction, part of the National Institutes of Health, completed a review of BPA in September 2008 and expressed “some concern” for effects on the brain, behavior, and prostate gland in fetuses, infants, and children at current human exposures to BPA. In the update of the draft assessment “BPA for Use in Food Contact Applications” in January 2010, the FDA shares at this interim stage the perspective of the National Toxicology Program that recent studies provide reason for some concern. The FDA also recognizes substantial uncertainties with respect to the overall interpretation of these studies and their potential implications for human health effects of BPA exposure. These uncertainties relate to issues such as the routes of exposure employed, the lack of consistency among some of the measured endpoints or results between studies, the relevance of some animal models to human health, differences in the metabolism (and detoxification) of and responses to BPA both at different ages and in different species, and limited or absent dose response information for some studies. The rating “some concern” about potential effects of BPA based on studies using novel approaches to test for subtle effects, which had been stated by the FDA/NHIES in 2008, will be addressed by a specific ongoing FDA research program. Regarding interim public health recommendations, the FDA supports reasonable steps to reduce exposure of infants to BPA in the food supply. In addition, the FDA will work with industry to support and evaluate manufacturing practices and alternative substances that could reduce exposure of the population and is supporting the industry's actions to stop producing BPA-containing bottles and infant feeding cups for the US market. The FDA is facilitating the development of alternatives to BPA for the linings of infant formula cans. But FDA still considers the TDI as valid.

### Japan

The human health risk assessment of the Japanese New Energy and Industrial Technology Development Organization (NEDO), the Research Center for Chemical Risk Management (CRM), and the National Institute of Advanced Industrial Science and Technology (AIST) from November 2007 concluded that both the human health risks and ecological risks posed by BPA were below the levels of concern. MOEs (margin of exposure) for the endpoints reduction in body weight gain, multinucleated giant hepatocytes in the liver, and reproductive toxicity were sufficiently large, even in the highest exposure group. Therefore, it will be unnecessary to prohibit or restrict the use of BPA at this time.

## Footnotes

1 The Advisory Committee of the German Society of Toxicology is elected by the members of the German Society of Toxicology and consists of representatives from academia, industry, and administration in order to guarantee a broad range of toxicological competence. The Advisory Committee may consult further experts with expertise in specific fields of Toxicology. In case of the present work, the committee included Dr. Wolfgang Völkel as an additional expert and sent the manuscript to Wolfgang Dekant, and Regine Kahl for discussion. The Advisory Committee presents and justifies its activities to the members of the German Society of Toxicology, for example at the yearly plenary meeting. The German Society of Toxicology is the largest scientific toxicological organization in Europe, with more than 1000 members..

2 See, for example, the criteria on reliability, relevance, and adequacy of data and studies by Klimisch et al., 1997.
